# Preparation of multifunctional nanobubbles and their application in bimodal imaging and targeted combination therapy of early pancreatic cancer

**DOI:** 10.1038/s41598-021-82602-9

**Published:** 2021-03-18

**Authors:** Hengli Yang, Ping Zhao, Yonggang Zhou, Qiaoying Li, Wenbin Cai, Zongxia Zhao, Jian Shen, Kechun Yao, Yunyou Duan

**Affiliations:** 1 Department of Ultrasound Diagnosis, Tang Du Hospital, Fourth Military Medical University, Xi’an, China; 2 Department of Ultrasound Diagnosis, The Second Affiliated Hospital, Xi’an Medical College, Xi’an, China; 3 Special Diagnosis Department, General Hospital of Tibet Military Command, Lhasa, China; 4 Department of Ultrasound Diagnosis, Air Force General Hospital, Beijing, China

**Keywords:** Cancer, Nanoscience and technology

## Abstract

Pancreatic cancer will gradually become the second leading cause of cancer death due to its poor suitability for surgical treatment, frequent recurrence and metastasis, and insensitivity to radiotherapy and chemotherapy. Strategies for precise early detection and effective targeted treatment of pancreatic cancer are urgently needed. Because of its unique advantages, molecular targeted contrast-enhanced ultrasound imaging (CEUI) has generated new opportunities to overcome this challenge. The aim of this study was to explore multifunctional nanobubbles named IR780-NBs-DTX as novel ultrasound contrast agents (UCAs) for dual-mode targeted imaging and photothermal ablation combined with chemotherapy for pancreatic cancer. An optimized “film hydration method” was used to prepare IR780-NBs-DTX in this research. The characteristics and ability of the new UCAs were detected via in vitro, in vivo and ex vivo experiments. The initial dose of 0.15 mg IR-780 iodide/1.0 mg DTX was considered to be the best formula for IR780-NBs-DTX, and the concentration of 6 ×10^6^ bubbles/mL was best for CEUI. The excellent characteristics of IR780-NBs-DTX, including a uniform nanoscale particle size (349.8± 159.1 nm, n= 3), good performance in dual-mode imaging, high stability and reliable biocompatibility, were also proven. In the in vitro cell experiments, IR780-NBs-DTX targeted more pancreatic cancer cells than the control treatments, and the targeting rate was approximately 95.6± 1.7%. Under irradiation with an 808 nm laser, most cells died. Furthermore, the in vivo study demonstrated that IR780-NBs-DTX could precisely detect pancreatic cancer through near infrared fluorescence (NIRF) imaging and CEUI, and the tumor almost disappeared at 18 days after combined treatment. In ex vivo experiments, immunohistochemistry (IHC) and immunofluorescence (IF) showed that the expression of HSP70 increased and that of PCNA decreased, and many apoptotic tumor cells were observed by TUNEL staining in the IR780-NBs-DTX group. The newly prepared IR780-NBs-DTX are novel nanosized UCAs with high efficiency for dual-mode molecular targeted imaging and combined therapy, and they may have future potential applications in the precise detection and effective targeted therapy of small and metastatic lesions in the early stage of pancreatic cancer.

## Introduction

Pancreatic cancer is currently the fourth leading cause of cancer death worldwide, and it has attracted increasing attention due to its poor prognosis. Most patients with pancreatic cancer have local infiltration and distant metastasis when they are diagnosed, and only 15–20% of patients can undergo radical surgery. Even then, because of the complex anatomical structure of the pancreas and its surrounding structures, the recurrence rate and mortality rate are very high after surgery^1^. In recent decades, chemotherapy and radiotherapy have been used to effectively inhibit the growth of malignant pancreatic cells, but the results have not significantly improved the survival rate of patients^2^. Late detection and tolerance to radiotherapy and chemotherapy are the main causes of poor prognosis in pancreatic cancer patients^3^. The median survival time of these patients is only 3–5 months, and the 5-year survival rate is less than 5%^2^. It is estimated that by 2020, pancreatic cancer will be the second leading cause of cancer death^4^. It is clear that the development of strategies for precise early detection and targeted treatment of pancreatic cancer is urgently needed^2^.

Molecular targeted imaging technology, which has been developed in recent years, shows promise for application in this context. At present, there are several mature molecular targeted imaging methods, such as PET, SPECT, MRI and photoacoustic imaging^5^. However, the limitations of these imaging technologies include radiation pollution and high costs. Recently, molecular targeted CEUI has attracted attention because of its advantages, including the lack of radiation pollution, real-time display, low costs and easy operation^6^. Therefore, it is important to develop molecular targeted UCAs with excellent performance for this technology. In past decades, microbubbles (MBs) loaded with antibodies, drugs and/or genes have been widely studied for use in intravascular molecular targeted CEUI and treatment of tumors^7–9^. However, MBs cannot pass through the tumor vascular endothelial gap (380–780 nm) and arrive at the target on tumor cells because of their large size (> 1 μm)^10^. Even then, the short lifespan of MBs in vivo is also an obstacle to their therapeutic effects in tumors^11^. To achieve real molecular imaging and effective treatment of tumors, stable nanosized ultrasound contrast agents are necessary. Recently, nanobubbles (NBs) prepared with fluorocarbon gas and coated with different shell membranes have been explored as UCAs^12^. NBs with small particle sizes can penetrate the pores of tumor vessels and accumulate in the tumor interstitium via the enhanced permeability and retention (EPR) effect. At the same time, compared with MBs, NBs have a prolonged retention time in the circulation in vivo^10^. However, it has been proven by some studies that NBs display poor tumor selectivity in vivo after intravenous injection because of clearance by the reticuloendothelial system (RES)^13,14^. The traditional strategy to overcome this issue is to use tumor-specific antibodies with an ultrasmall size and high affinity to conjugate NBs^15–17^. However, it is difficult to identify appropriate antibodies, and most of the techniques for combining antibodies and NBs rely on tedious chemical steps that may reduce the stability of NBs.

IR-780 iodide, a prototypic NIR heptamethine cyanine agent, is a targeting element that seems to be mediated by organic-anion-transporting polypeptides (OATPs) overexpressed in cancer cells^18^. Furthermore, an increasing number of studies have verified that IR-780 iodide is effective in high-intensity NIRF imaging, with spontaneous accumulation in tumors and the ability to mediate cancer photothermal therapy (PTT) and photodynamic therapy (PDT)^19^. However, the characteristics of IR-780 iodide, such as poor water solubility, rapid clearance, and acute toxicity, limit its direct clinical use^19,20^. A good approach is to encapsulate IR-780 iodide in nanostructures and deliver it to tumor cells^21–23^. Notably, carrying IR-780 iodide within nanostructures does not require chemical bond modification, and the combination is convenient and reliable. In our previous study, we loaded IR-780 iodide into NBs and then preliminarily verified that the conjugates could target female tumor cells and glioma cells and performed NIRF and CEUI imaging and photothermal ablation of tumors for 15 days^18,24^.

**Figure 1:**
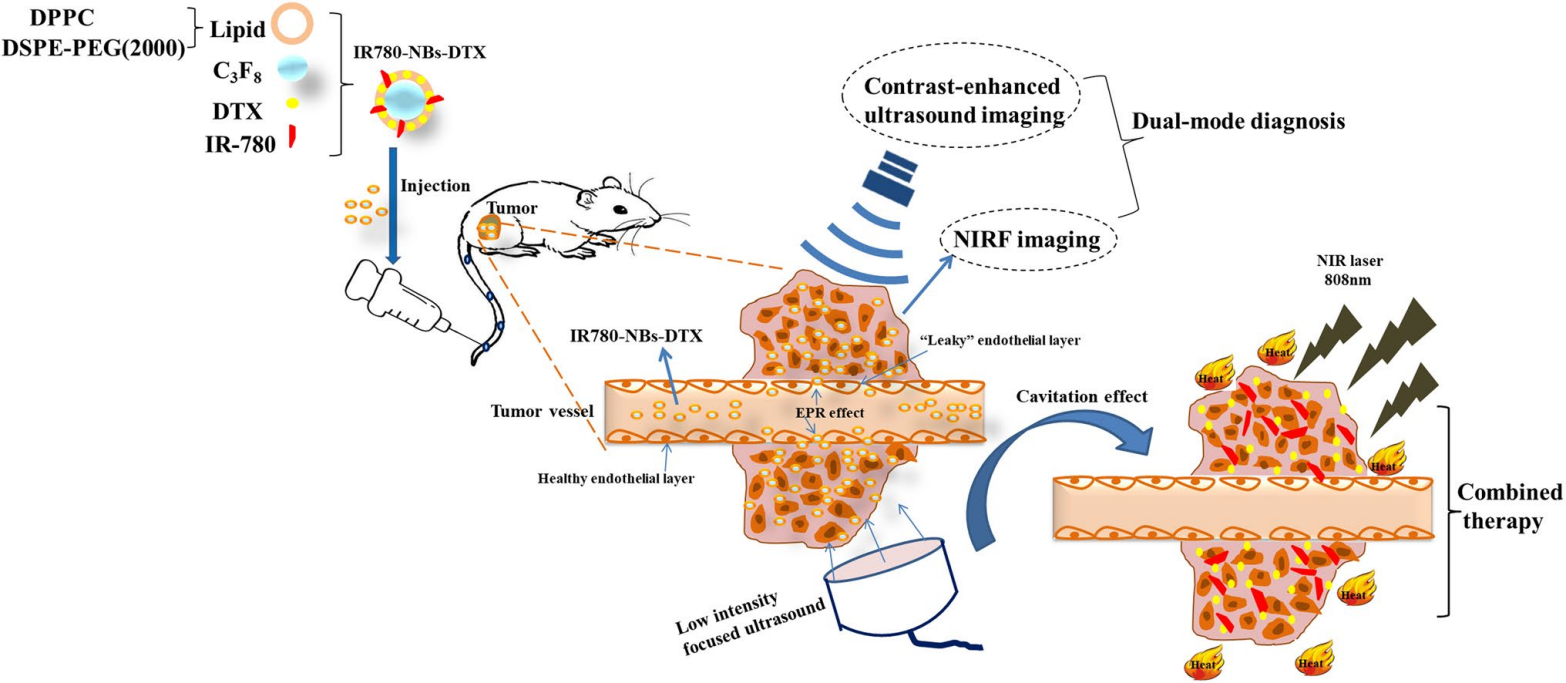
Schematic illustration of IR780-NBs-DTX preparation, molecular targeted imaging and combined treatment of tumors.

To further improve the killing effect for tumor cells, in this study, IR-780 iodide and docetaxel (DTX) were simultaneously loaded into the lipid shells of NBs to prepare novel UCAs named IR780-NBs-DTX for dual-mode molecular targeted imaging and targeted combination therapy of pancreatic cancer (Fig. 1). Docetaxel (DTX), one of the most important chemotherapeutic agents, has been widely used for the treatment of various types of cancers. However, in the clinic, chemotherapy with DTX results in many undesirable side effects due to the usage of organic solvents in the injection and due to its low selectivity for tumor cells^25^. Theoretically, loading DTX into NBs will greatly promote the therapeutic effects on tumors and decrease side effects. In this research, the procedure for preparing IR780-NBs-DTX was further optimized, and the new IR780-NBs-DTX had good characteristics and biosafety. In vitro and in vivo experiments verified that IR780-NBs-DTX have the potential advantages of accurate detection and targeted combination therapy for pancreatic cancer.

## Results

### Exploration of the appropriate entrapment of IR-780 iodide and DTX in IR780-NBs-DTX

**Figure 2:**
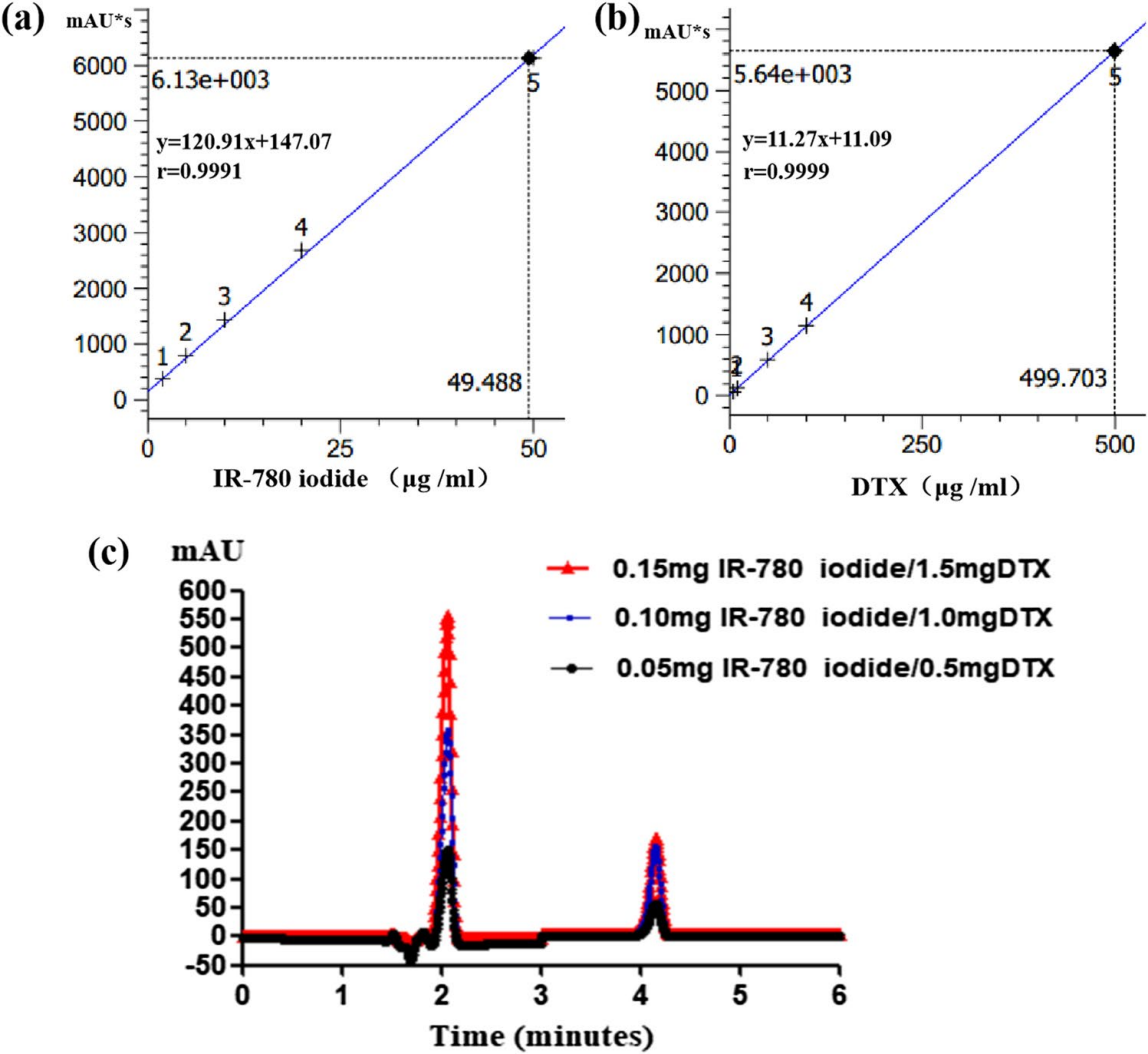
The entrapment of IR-780 iodide and DTX in IR780-NBs-DTX with different initial doses. (**a**,**b**) The standard curves of IR-780 iodide and DTX obtained via HPLC. (**c**) The signal intensity of IR-780 iodide at 2.1 min and that of DTX at 4.1 min with different initial doses via HPLC.

First, standard curves of IR-780 iodide and DTX were generated (Fig. 2a,b). Over a gradient of drug doses, the entrapment of both drugs was detected using HPLC. In the different dose groups, the signals were detected at approximately 2.1 min for IR-780 iodide and at 4.1 min for DTX. The signals of IR-780 iodide and DTX in the 0.15 mg IR-780 iodide/1.0 mg DTX group were obviously higher than those in the other groups (Fig. 2c). As shown in Table 1, the EE and DL of IR-780 iodide and DTX were measured. For IR-780 iodide, as the initial dose increased from 0.05 to 0.10 mg and 0.15 mg, the DL increased from 0.37 ± 0.06% to 1.29 ± 0.55% (P < 0.05); in contrast, the EE increased at 0.10 mg (61.5 ± 0.9% vs. 51.6 ± 1.2%, P < 0.05) and then decreased at 0.15 mg, but the difference was not statistically significant (61.5 ± 0.9% vs*.* 60.4 ± 0.5%, P > 0.05). For DTX, as the initial dose increased from 0.5 to 1.0 mg and 1.5 mg, the DL increased from 1.7 ± 0.2% to 4.6 ± 0.9% (P < 0.05) and 4.9 ± 0.7%; in contrast, the EE also increased at 1.0 mg and then decreased at 1.5 mg, and the difference was statistically significant (32.5 ± 1.9% vs*. 23*.8 ± 2.1%, P < 0.05; 32.5 ± 1.9% vs*.* 22.9 ± 3.2%, P < 0.05). Thus, entrapment of 0.15 mg IR-780 iodide/1.0 mg DTX was considered to be a better formula for subsequent experiments.

**Table 1:** The EE and DL of IR-780 iodide and DTX in IR780-NBs-DTX with different initial doses.

Drug	Initial adding dose (mg)	EE	DL
IR-780 iodide	0.05	51.6 ± 1.2%	0.37 ± 0.06%
	0.10	61.5 ± 0.9%^¥^	0.88 ± 0.09%
	0.15	60.4 ± 0.5%	1.29 ± 0.55%^#^
DTX	0.05	23.8 ± 2.1%	1.7 ± 0.2%
	0.10	32.5 ± 1.9%^&,^^	4.6 ± 0.9%*
	0.15	22.9 ± 3.2%	4.9 ± 0.7%

^#^P < 0.05, significantly different from the DL of IR-780 iodide with an initial dose of 0.05 mg. ^¥^P < 0.05, significantly different from the EE of IR-780 iodide with an initial dose of 0.05 mg. *P < 0.05, significantly different from the DL of DTX with an initial dose of 0.5 mg. ^&^P < 0.05, significantly different from the EE of DTX with an initial dose of 0.5 mg. ^^^P < 0.05, significantly different from the EE of DTX with an initial dose of 1.5 mg. Data are presented as the mean ± standard deviation.

### CEUI of IR780-NBs-DTX in vitro

**Figure 3:**
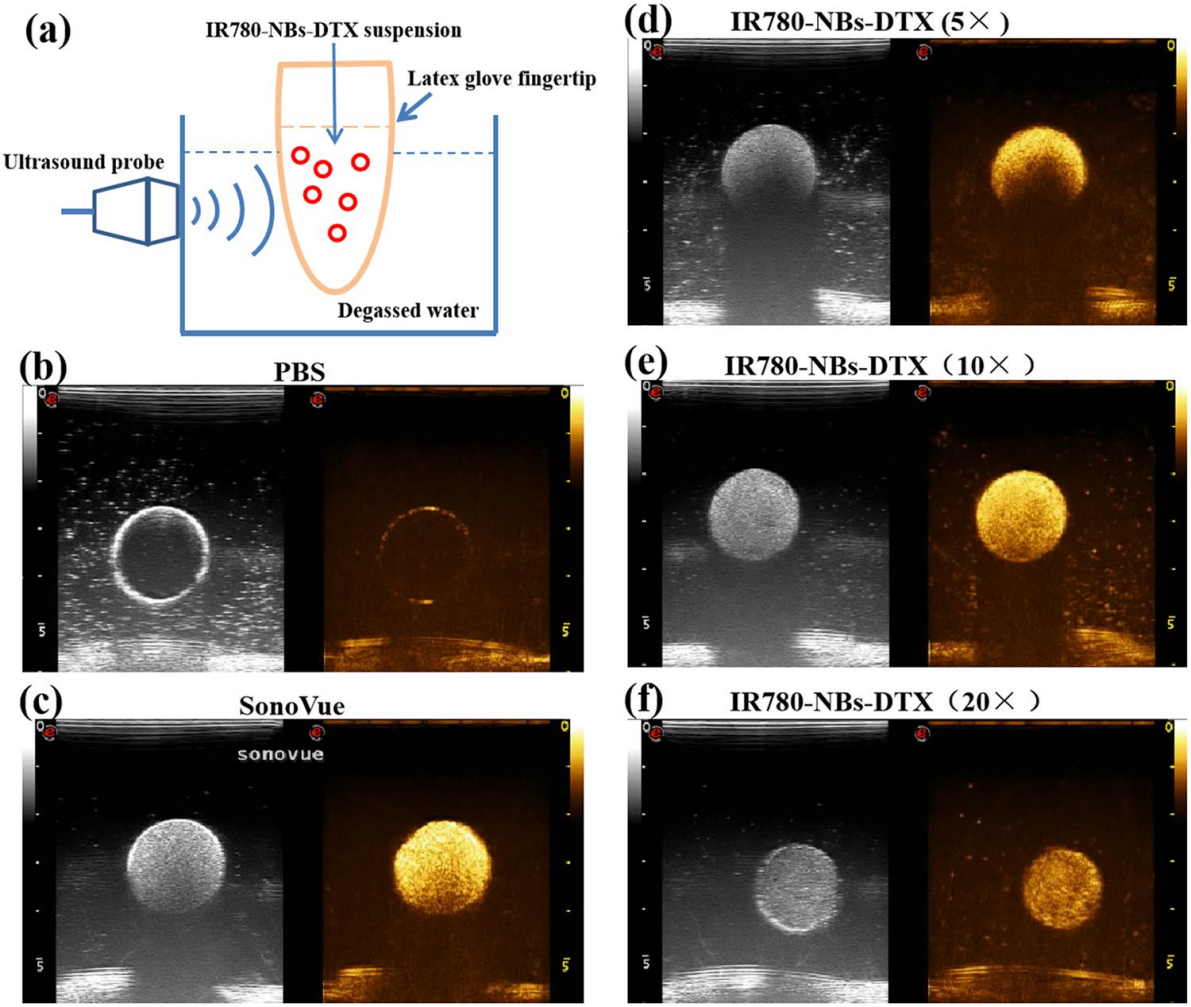
The CEUI of IR780-NBs-DTX in vitro. (**a**) Schematic illustration of the analytical setup for CEUI of IR780-NBs-DTX in vitro. (**b**,**c**) The CEUI of PBS and SonoVue as controls. (**d**,**e**,**f**) CEUI of different concentrations of IR780-NBs-DTX (×5: 1.2 × 10^7^ bubbles/mL; ×10: 6 × 10^6^ bubbles/mL; ×20: 3 × 10^6^ bubbles/mL).

As shown in Fig. 3, PBS had no obvious ultrasound-enhancing effect on imaging, whereas SonoVue, which was used as a positive control, showed good echogenicity. The ultrasound echogenicity of IR780-NBs-DTX (with initial addition of 0.15 mg IR-780 iodide/1.0 mg DTX) was observed at three different concentrations. Interestingly, the concentration of 6 × 10^6^ bubbles/mL performed well in ultrasound-enhanced imaging, similar to SonoVue; however, concentrations of both 1.2 × 10^7^ bubbles/mL and 3 × 10^6^ bubbles/mL resulted in weaker echogenicity. IR780-NBs-DTX at a concentration of 6 × 10^6^ bubbles/mL was obviously better for subsequent experiments.

### Characteristics of IR780-NBs-DTX

**Figure 4:**
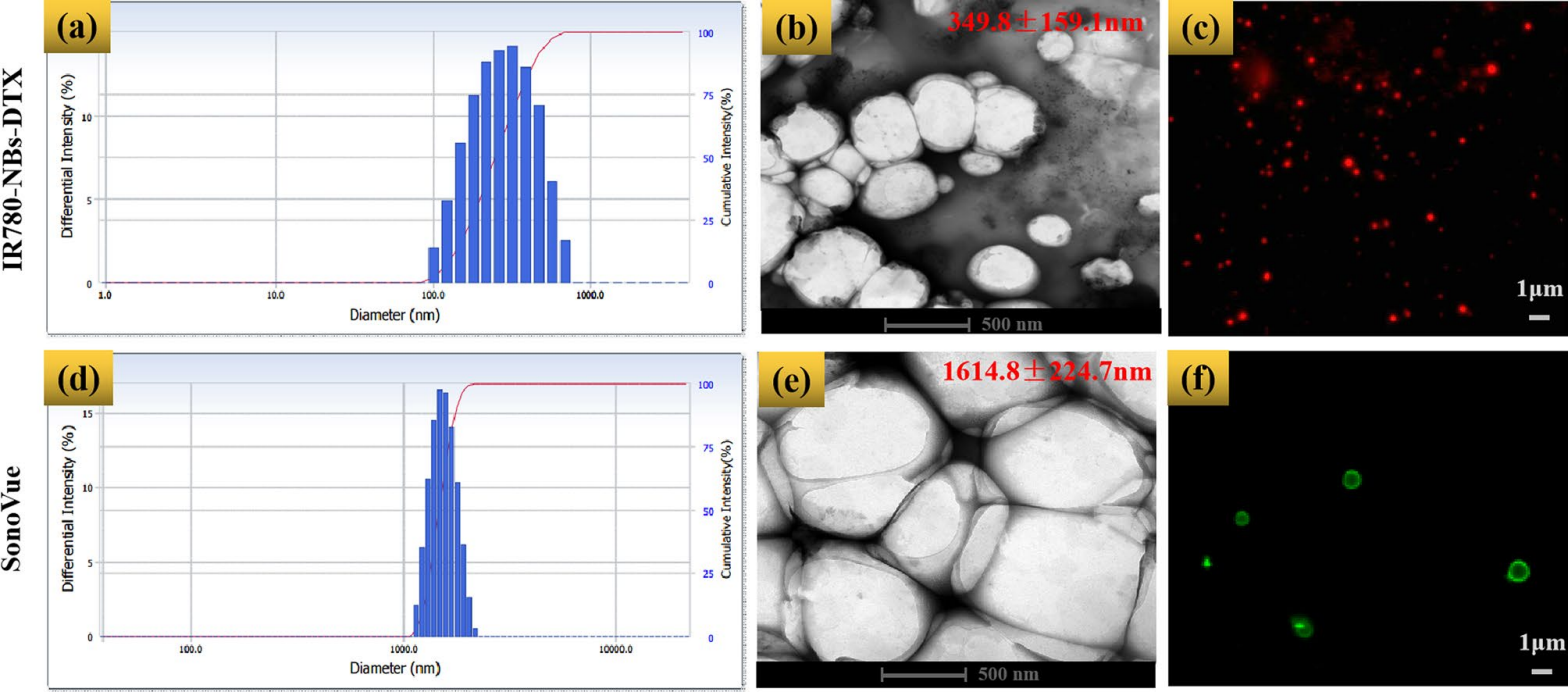
The characteristics of IR780-NBs-DTX and SonoVue. (**a**,**d**) The intensity distribution of IR780-NBs-DTX and SonoVue. (**b**,**e**) IR780-NBs-DTX and SonoVue were assessed via TEM. (**c**,**f**) IR780-NBs-DTX and SonoVue were detected by CLSM.

As shown in Fig. 4, the average particle size of IR780-NBs-DTX was determined to be 349.8 ± 159.1 nm, with a polydispersity index (P.I.) = 0.190 ± 0.058 (n = 3) (Fig. 4a), whereas that of SonoVue was 1614.8 ± 224.7 nm with a P.I. = 0.142 ± 0.078 (n = 3) (Fig. 4d). Under TEM (Fig. 4b,e), both IR780-NBs-DTX and SonoVue had spherical shapes; nevertheless, the size of IR780-NBs-DTX was obviously smaller than that of SonoVue. CLSM also verified that IR780-DTX-NBs with a smaller size took on NIRF (Fig. 4c), which means that IR-780 iodide was successfully loaded in the NBs. At the same time, SonoVue stained with Dio exhibited green fluorescence, and the size distribution was larger than 1 μm (Fig. 4f).

### Stability, biocompatibility and photothermal effect of IR780-NBs-DTX

**Figure 5:**
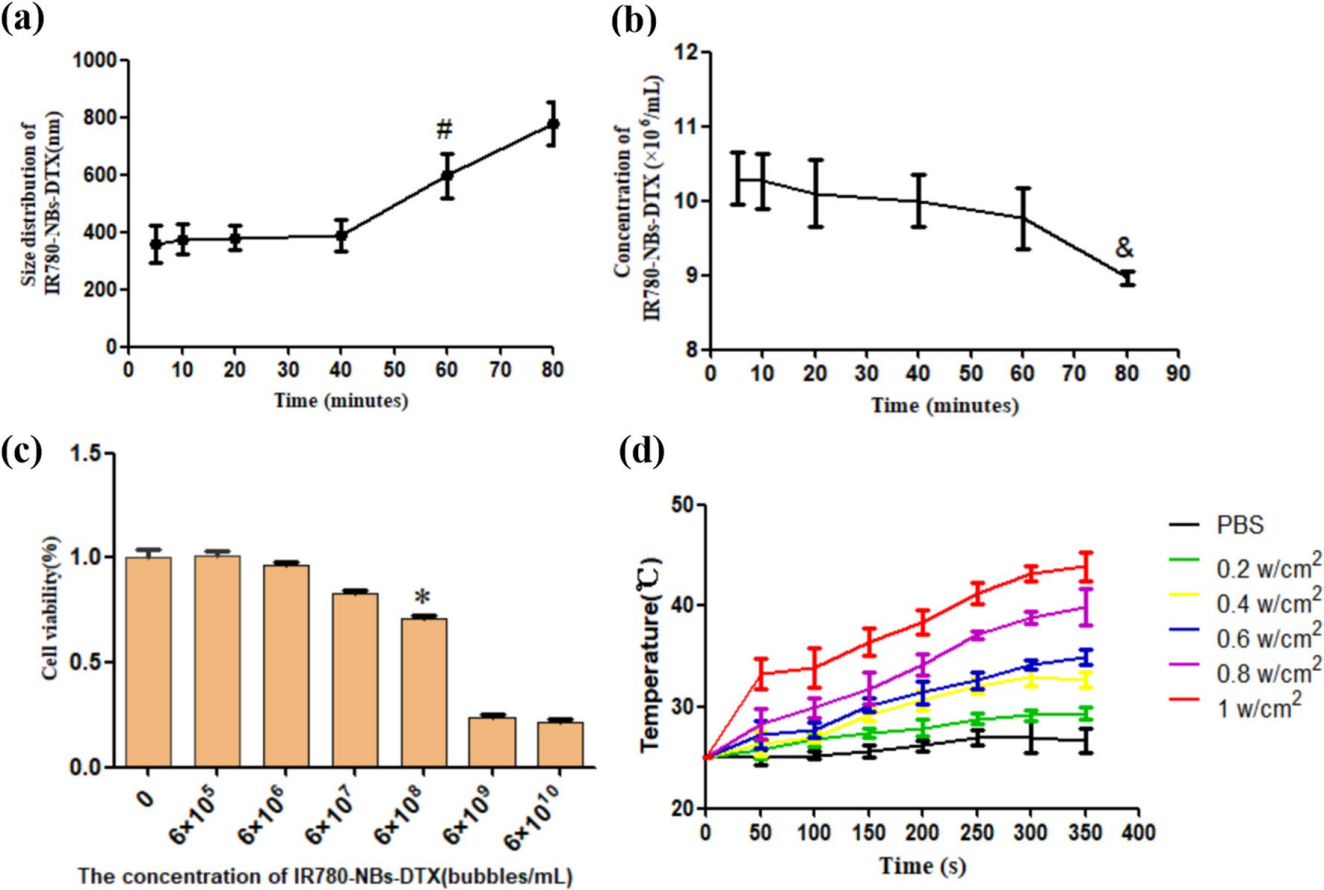
The stability, biocompatibility and photothermal effect of IR780-NBs-DTX. (**a**,**b**) Changes in the size distribution and concentration of IR780-NBs-DTX over 80 min. ^#^P < 0.05, significantly different from the size distribution at 5 min; ^&^P < 0.05, significantly different from the concentration at 5 min. (**c**) The cytotoxicity of IR780-NBs-DTX at different concentrations (0: control cells; 6 × 10^5^/mL; 6 × 10^6^/mL; 6 × 10^7^/mL; 6 × 10^8^/mL; 6 × 10^9^/mL; 6 × 10^10^/mL). *P < 0.05, significantly different from the cytotoxicity in control cells. (**d**) The photothermal effect of IR780-NBs-DTX under an 808 nm laser at different intensities.

The stability of IR780-NBs-DTX was assessed by the changes in size distribution and concentration. At 40 min, the size distribution of IR780-NBs-DTX showed no obvious change compared with that at 5 min (386.7 ± 63.2 nm vs*.* 359.3 ± 83.2 nm, P > 0.05), but at 60 min, the size of IR780-NBs-DTX increased significantly (596.7 ± 98.6 nm vs*.* 359.3 ± 83.2 nm, P < 0.05) (Fig. 5a). In addition, at 60 min, the concentration of IR780-NBs-DTX was more stable than that at 5 min ((9.8 ± 0.7) × 10^6^ bubbles/mL *vs.* (10.3 ± 0.5) × 10^6^ bubbles/mL, P > 0.05); however, at 80 min, the concentration of IR780-NBs-DTX decreased ((8.9 ± 0.2) × 10^6^ bubbles/mL vs*.* (10.3 ± 0.5) × 10^6^ bubbles/mL, P < 0.05) (Fig. 5b).

The biocompatibility of IR780-NBs-DTX is also an important factor. In Fig. 5c, the cytotoxicity of IR780-NBs-DTX at different concentrations was detected. When the concentration increased to 6 × 10^6^ bubbles/mL, there was a significant decrease in cell viability (71.2 ± 1.2% vs*.* 99.8 ± 2.1%, P < 0.05).

Then, the photothermal effect of IR-780 iodide loading in IR780-NBs-DTX was detected at the same concentration. As the 808 nm laser intensity changed from 0.2, 0.4, 0.6, and 0.8 to 1 w/cm^2^, the temperature of the IR780-NB-DTX solution showed a stepped increase for 300 s and plateaued at 350 s in the different groups. In the 1 w/cm^2^ group, the temperature showed the greatest increase. However, the temperature of PBS showed no obvious increase (Fig. 5d).

### Tumor-targeting capability of IR780-NBs-DTX on pancreatic cancer cells in vitro

**Figure 6:**
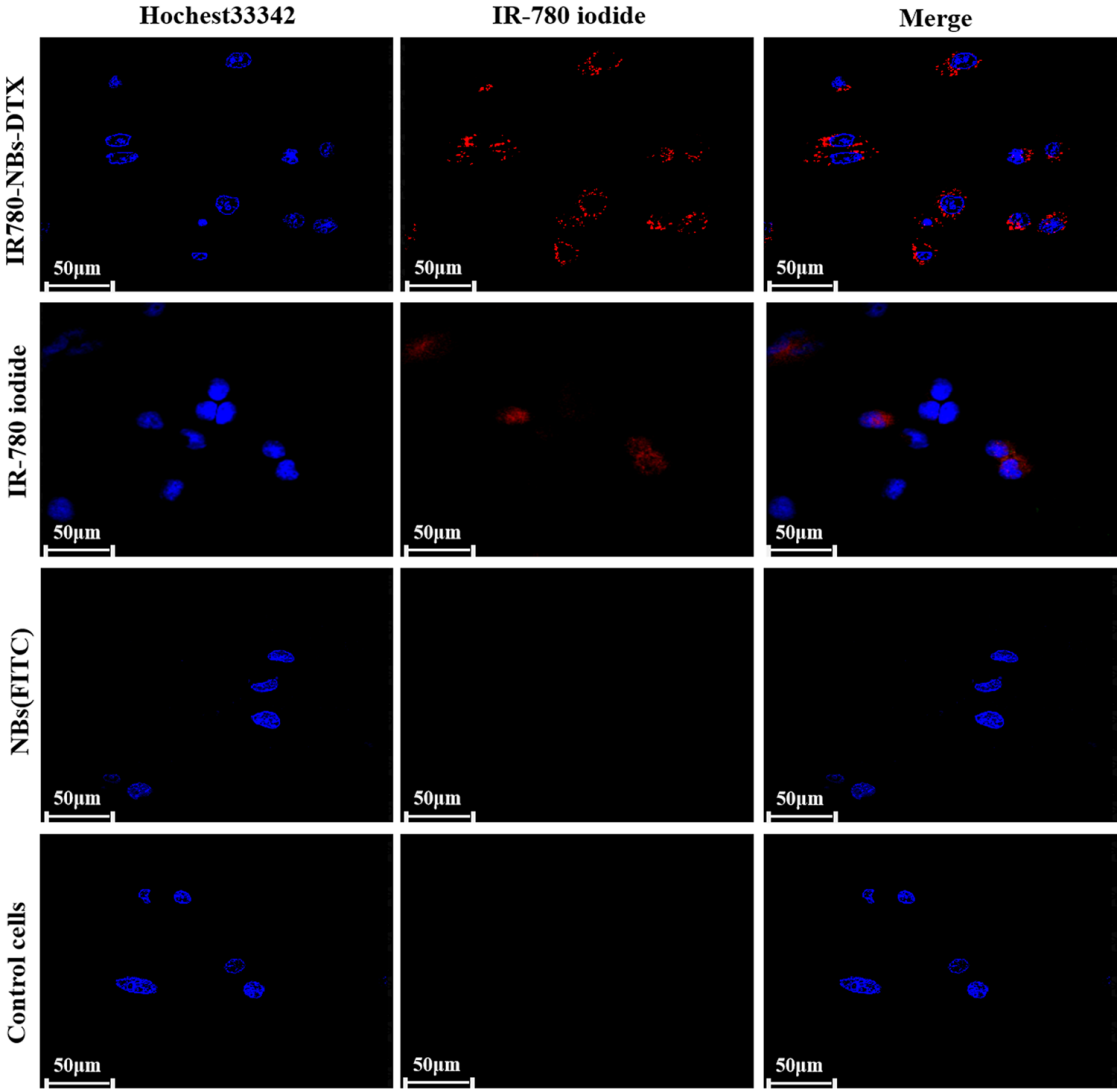
Tumor-targeting capability of IR780-NBs-DTX, IR-780 iodide and NBs(FITC) assessed via CLSM after incubation with Mia-Paca2 pancreatic cancer cells in vitro.

**Figure 7:**
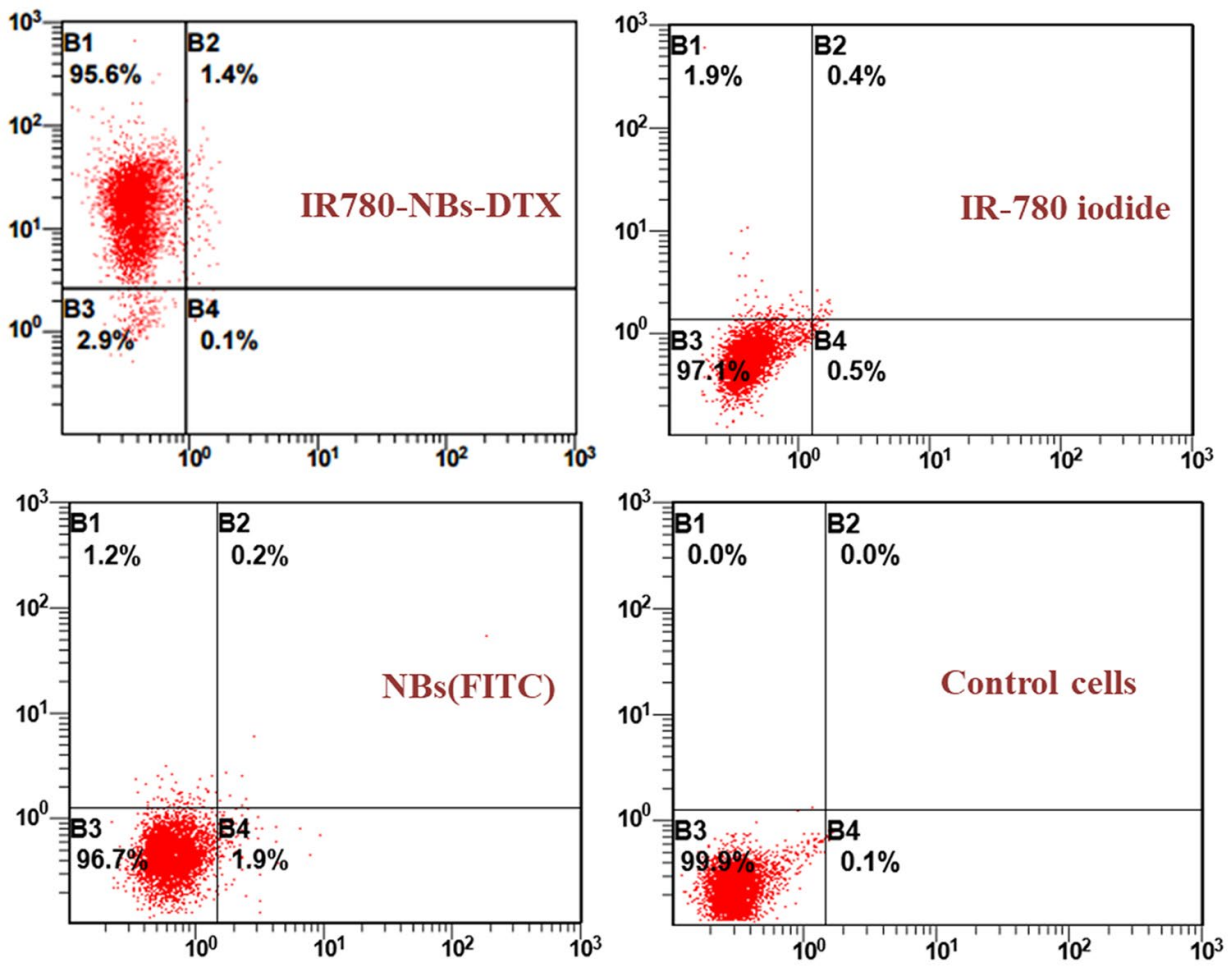
Tumor-targeting rates of IR780-NBs-DTX, IR-780 iodide and NBs(FITC) evaluated via FCM analysis after incubation with Mia-Paca2 pancreatic cancer cells.

The fluorescence levels on the Mia-Paca2 cells reflected the number of NBs targeted to the cells via CLSM. As shown in Fig. 6, there was no obvious fluorescence from the tumor cells in the NBs (FITC) group and a small amount of NIRF from tumor cells in the IR-780 iodide group. Interestingly, in the IR780-NBs-DTX group, a high level of NIRF was observed in tumor cells, which indicated that most of the IR780-NBs-DTX targeted the tumor cells. As shown in Fig. 7, FCM revealed that approximately 95.6 ± 1.7% of tumor cells exhibited NIRF in the IR780-NBs-DTX group, whereas 1.9 ± 0.4% of tumor cells exhibited NIRF in the IR-780 iodide group. In the NBs (FITC) group and the control cells group, there was almost no fluorescence in the tumor cells. The targeting rate of IR780-NBs-DTX to Mia-Paca2 cells was obviously higher than that of IR-780 iodide alone, and the difference was statistically significant (95.6 ± 1.7% vs*.* 1.9 ± 0.4%, P < 0.05).

### Effect of photothermal ablation combined with chemotherapy with IR780-NBs-DTX on pancreatic cancer cells in vitro

**Figure 8:**
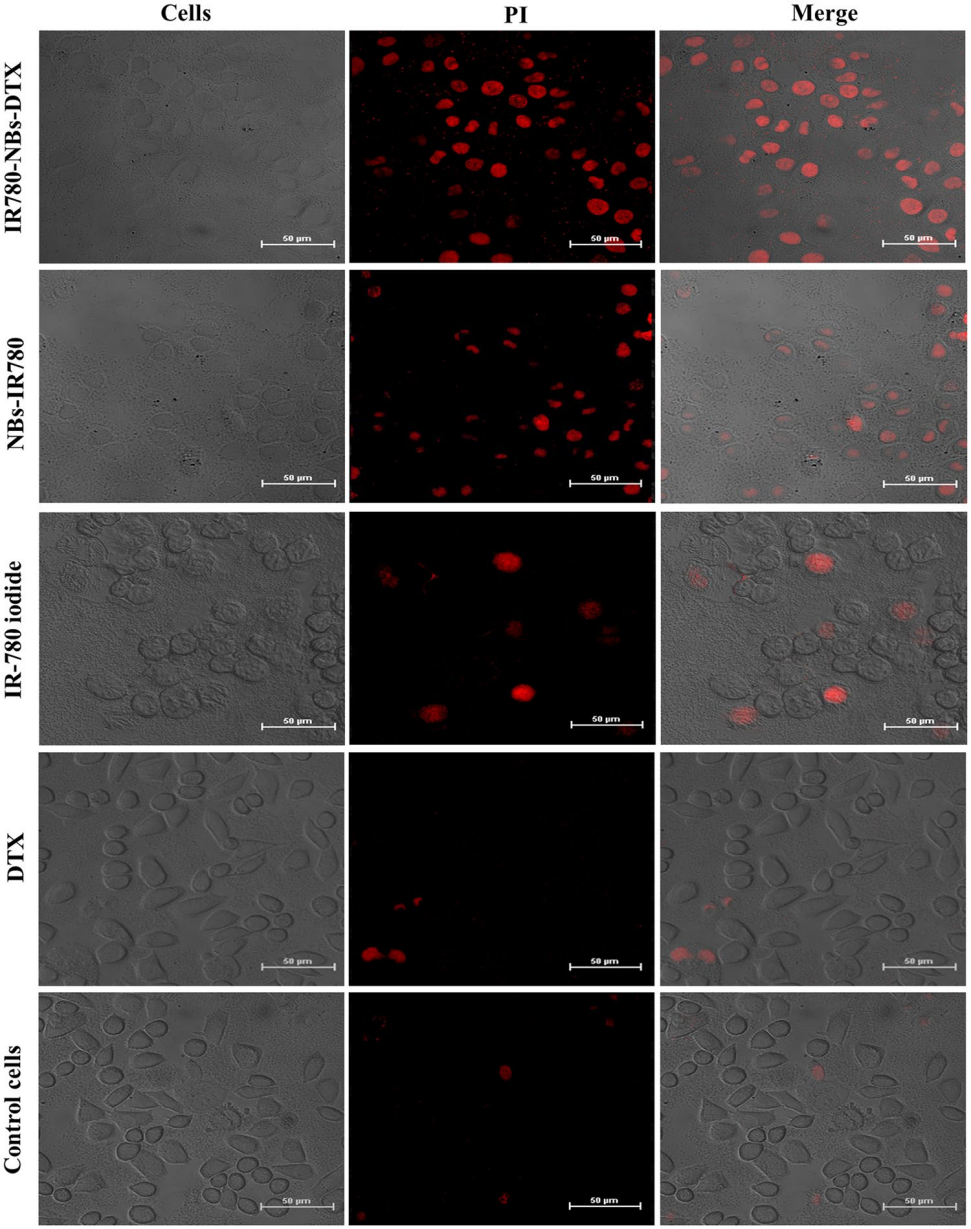
Photothermal ablation combined with chemotherapy with IR780-NBs-DTX, NBs-IR780, IR-780 iodide and DTX assessed via CLSM in Mia-Paca2 pancreatic cancer cells in vitro.

As shown in Fig. 8, in the IR780-NBs-DTX group and NBs-IR780 group, many tumor cells were stained with PI to detect apoptosis, and the former group had more apoptotic cells than the latter. In the IR-780 iodide group and DTX group, there were a few apoptotic cells stained with PI, and most of the tumor cells grew well in the visual field. In the control cells group, few apoptotic cells were detected, and all of the tumor cells grew well.

### CEUI of IR780-NBs-DTX on heterotopic subcutaneously transplanted pancreatic cancer in vivo

**Figure 9:**
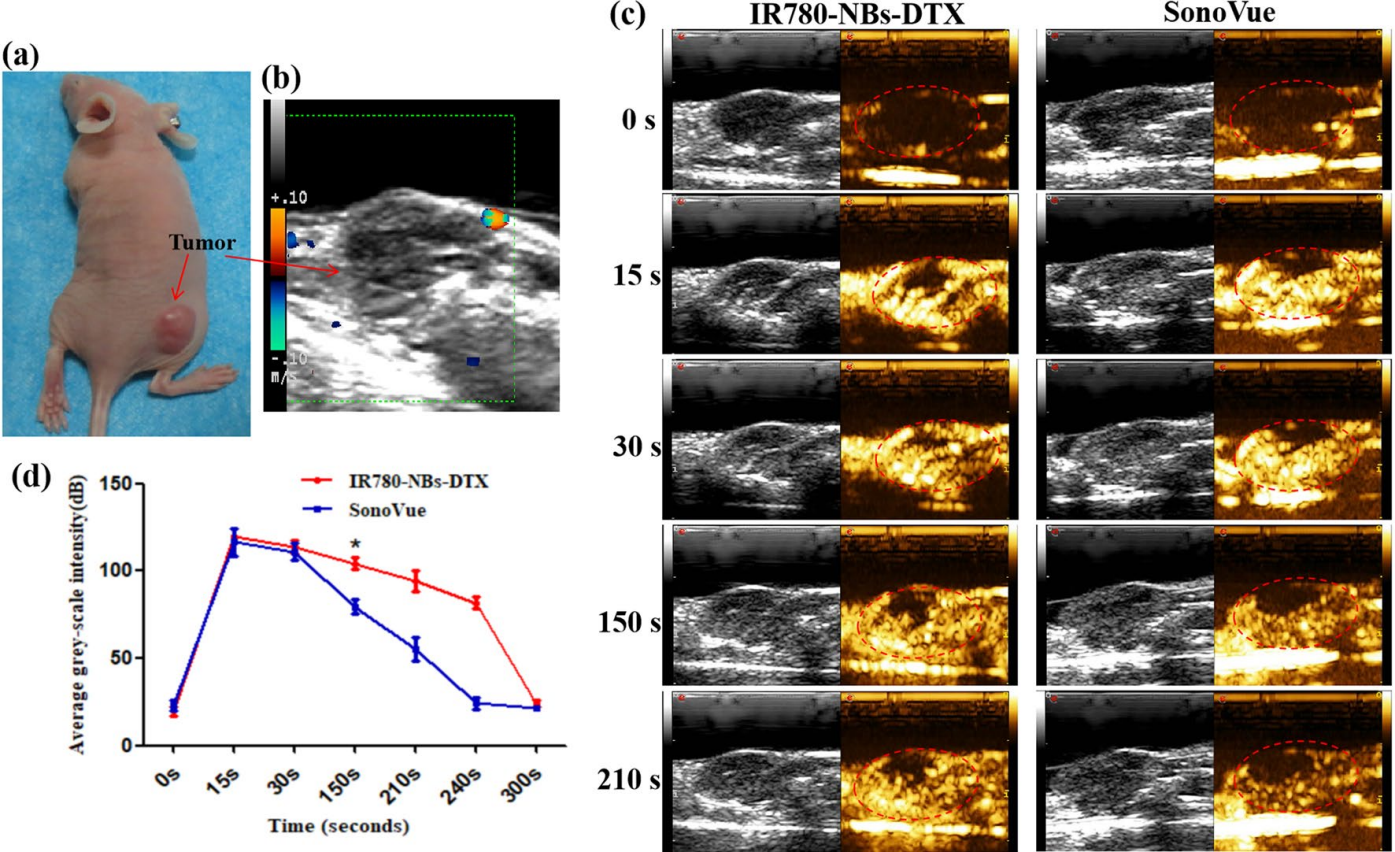
CEUI of IR780-NBs-DTX on subcutaneous xenotransplanted pancreatic cancer in vivo. (**a**) Nude mice bearing subcutaneous xenotransplanted pancreatic cancer. (**b**) Routine ultrasound for pancreatic cancer. (**c**) CEUI of pancreatic cancer mediated by IR780-NBs-DTX and SonoVue. The red dotted lines represent the tumor outline. (**d**) Analysis of the time-dependent echo intensity of CEUI mediated by IR780-NBs-DTX and SonoVue in pancreatic cancer. *P < 0.05, significantly different from the echo intensity of CEUI mediated by SonoVue at 150 s.

As shown in Fig. 9a, nude mice bearing subcutaneous xenotransplanted pancreatic cancer were examined by routine ultrasound, and the tumor boundary was not clear (Fig. 9b). Under CEUI (Fig. 9c), the selective imaging capability of IR780-NBs-DTX was detected. SonoVue was included as the control. In the early stage of CEUI, both UCAs had almost the same ultrasound enhancement intensity. However, 30 s later, although the echogenicity decreased in both groups, IR780-NBs-DTX continued to exhibit stronger echogenicity than SonoVue. Until 240 s, the echogenicity of IR780-NBs-DTX was greater than that of SonoVue. The comparison of the ultrasound enhancement intensity induced by IR780-NBs-DTX and SonoVue in vivo is shown in Fig. 9d. The time-intensity curve analysis indicated that the ultrasound enhancement intensity of IR780-NBs-DTX was significantly stronger than that of SonoVue from 150 s (104.157 ± 6.0 dB vs. 79.438 ± 7.0 dB, P < 0.05).

### Tumor-specific targeting and NIRF imaging capability of IR780-NBs-DTX in xenotransplanted pancreatic cancer in vivo

**Figure 10:**
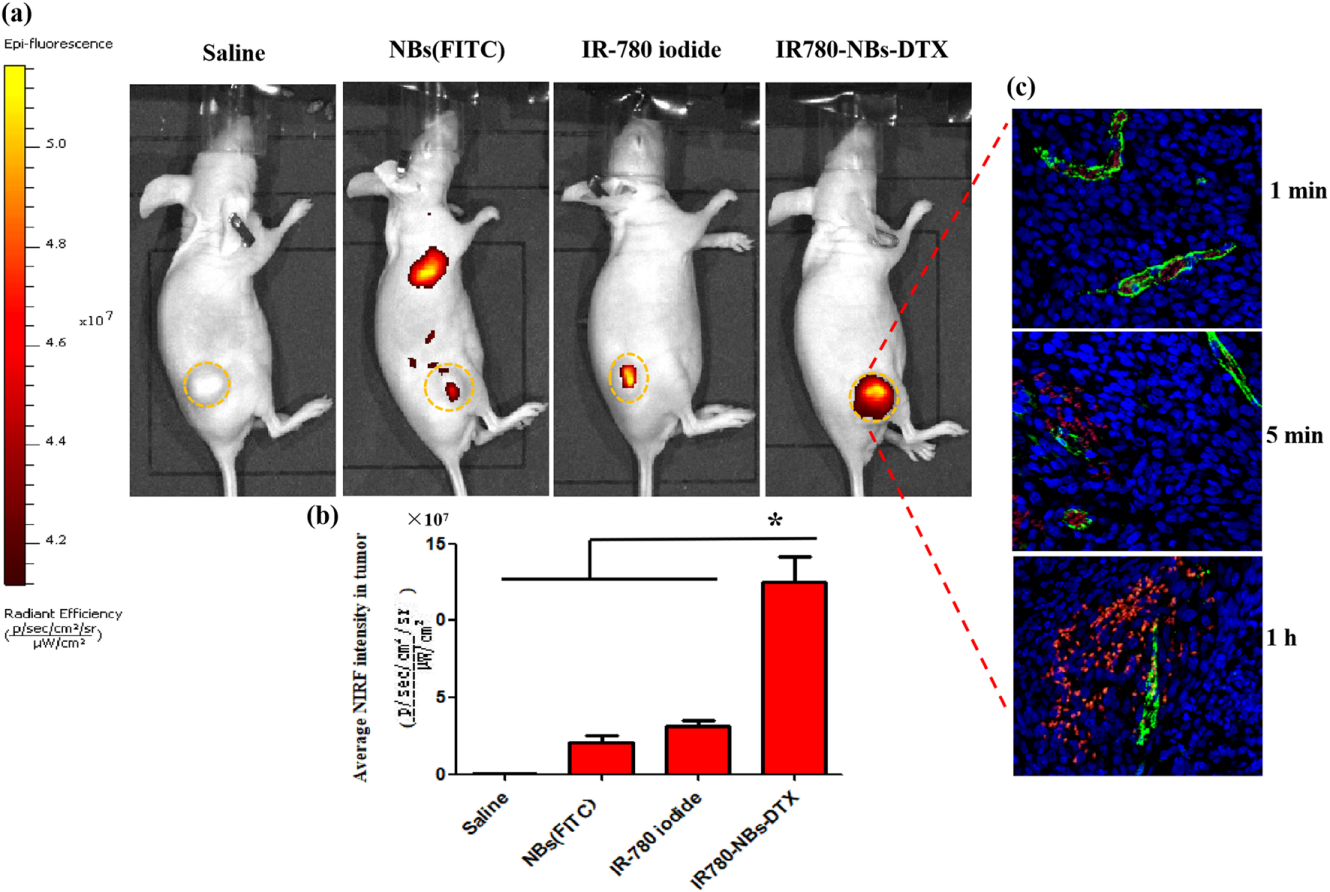
Tumor-specific targeting and NIRF imaging of IR780-NBs-DTX in subcutaneous xenotransplanted pancreatic cancer in vivo. (**a**) Nude mice bearing tumors derived from the Mia-Paca2 cell line were detected via the IVIS Lumina II system at 1 h after being injected with different contrast agents. The yellow dotted lines represent the tumor outline. (**b**) Comparison of the average fluorescence intensity of tumors in different groups. *P < 0.05, significantly different from the average fluorescence intensity of tumors in the saline group, NBs (FITC) group and IR-780 iodide group. (**c**) Immunofluorescence detection of tumor tissues for the IR780-NBs-DTX” group at 1 min, 5 min and 1 h after injection of contrast agents through the caudal vein.

As shown in Fig. 10a, at 1 h after injection of different contrast agents through the caudal vein, NIRF imaging of the tumor site was performed in nude mice injected with IR780-NBs-DTX and IR780 iodide, and the contrast agents were shown to accumulate in the tumors in all groups except the saline group. Interestingly, most IR780-NBs-DTX accumulated at the tumor site, whereas a small amount of NBs (FITC) and IR780-iodide accumulated at the tumor site. Furthermore, the average fluorescence intensity of tumors in different groups was analyzed statistically (Fig. 10b). The average fluorescence intensity of the IR780-NBs-DTX group was the obviously higher than that of the other groups, and the difference was statistically significant ((5.12 ± 0.69) × 10^7^ vs*.* (1.06 ± 0.23) × 10^7^, (5.12 ± 0.69) × 10^7^ vs*.* (1.54 ± 0.42) × 10^7^, and (5.12 ± 0.69) × 10^7^ vs*.* (2.98 ± 0.34) × 10^7^, P < 0.05).

Moreover, to more accurately observe the targeting and diffusion of IR780-NBs-DTX at the tumor site, immunofluorescence detection of tumor tissues was performed. In Fig. 10c, after injection through the caudal vein, all IR780-NBs-DTX accumulated in tumor vessels at 1 min. Then, 5 min later, some IR780-NBs-DTX leaked out of the tumor vessels and accumulated in the interstitial space of the cancer cells. Even then, most IR780-NBs-DTX were observed in the interstitial spaces of the cancer cells at 1 h.

### The biodistribution of IR780-NBs-DTX ex vivo

**Figure 11:**
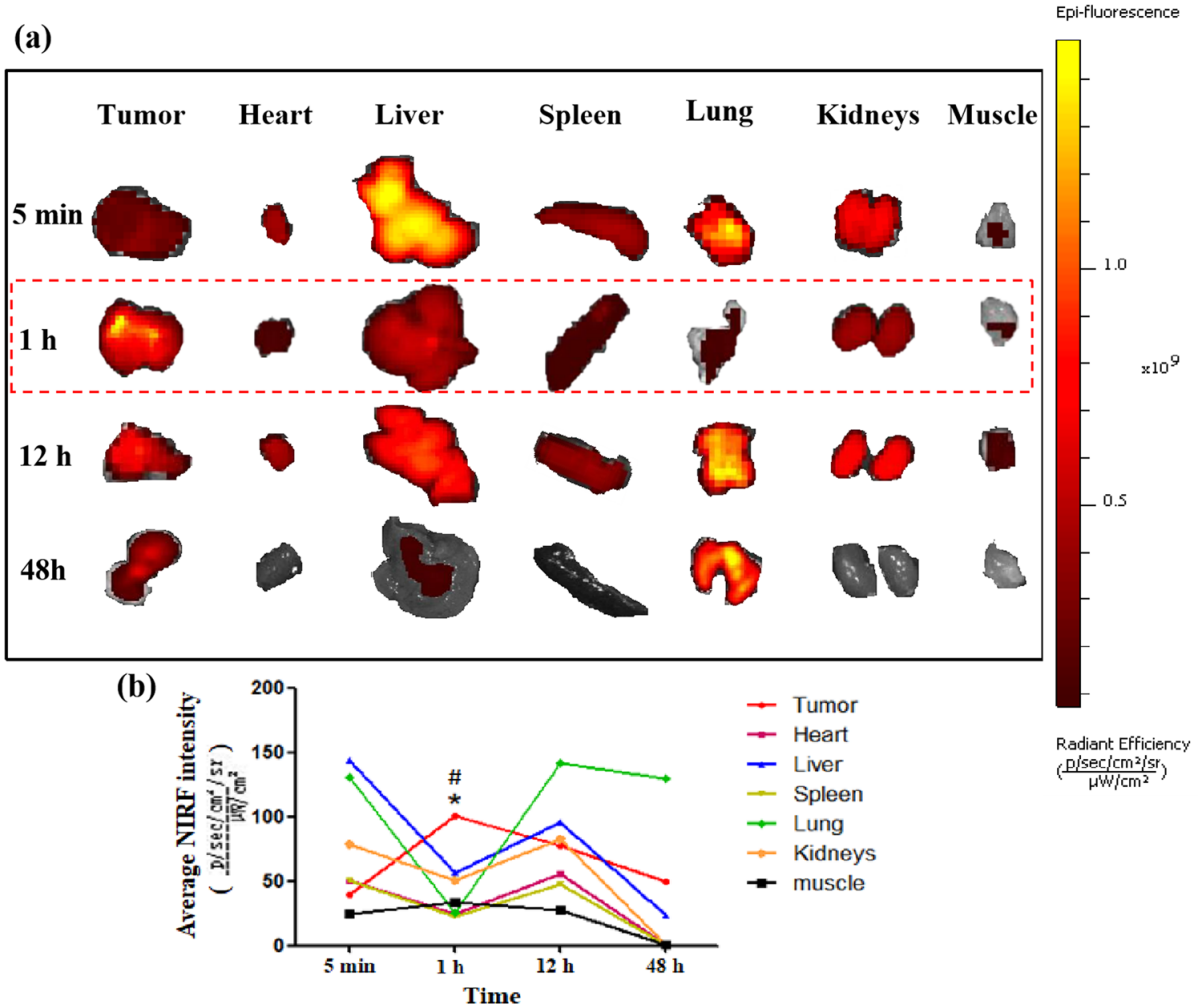
The biodistribution of IR780-NBs-DTX ex vivo via an IVIS Lumina II imaging station. (**a**) NIRF imaging of the tumor, heart, liver, spleen, lung, kidneys and muscle at 5 min, 1 h, 12 h and 48 h. (**b**) Analysis of the average NIRF intensity of the tumor, heart, liver, spleen, lung, kidneys and muscle at different time points. *P < 0.05, significantly different from the average NIRF intensity of tumors at 5 min, 12 h and 48 h. ^#^P < 0.05, significantly different from the average NIRF intensity of the liver, lung and kidneys at 1 h.

In Fig. 11a, there was a little NIRF intensity in tumors at 5 min, whereas the NIRF intensity rose to its highest level at 1 h and then decreased gradually at 12 h and 48 h. Compared with 5 min, 12 h and 48 h, the NIRF intensity of tumors at 1 h had obvious statistical significance ((1.02 ± 0.033) × 10^9^ vs*.* (0.38 ± 0.024) × 10^9^, (1.02 ± 0.033) × 10^9^ vs*.* (0.78 ± 0.022) × 10^9^, (1.02 ± 0.033) × 10^9^ vs*.* (0.50 ± 0.023) × 10^9^, P < 0.05)(Fig. 11b). Furthermore, in addition to targeting tumors, NIRF signals were detected mainly in the liver, lungs and kidneys. In the muscle, there was little NIRF accumulation (Fig. 11a). Nevertheless, at 1 h, the NIRF intensity was obviously higher than that of the liver, lungs and kidneys ((1.02 ± 0.033) × 10^9^ vs*.* (0.55 ± 0.016) × 10^9^, (1.02 ± 0.033) × 10^9^ vs*.* (0.25 ± 0.019) × 10^9^, (1.02 ± 0.033) × 10^9^ *vs.* (0.51 ± 0.021) × 10^9^, P < 0.05) (Fig. 11b).

### Photothermal ablation combined with chemotherapy of xenotransplanted pancreatic cancer mediated by IR780-NBs-DTX in vivo

**Figure 12:**
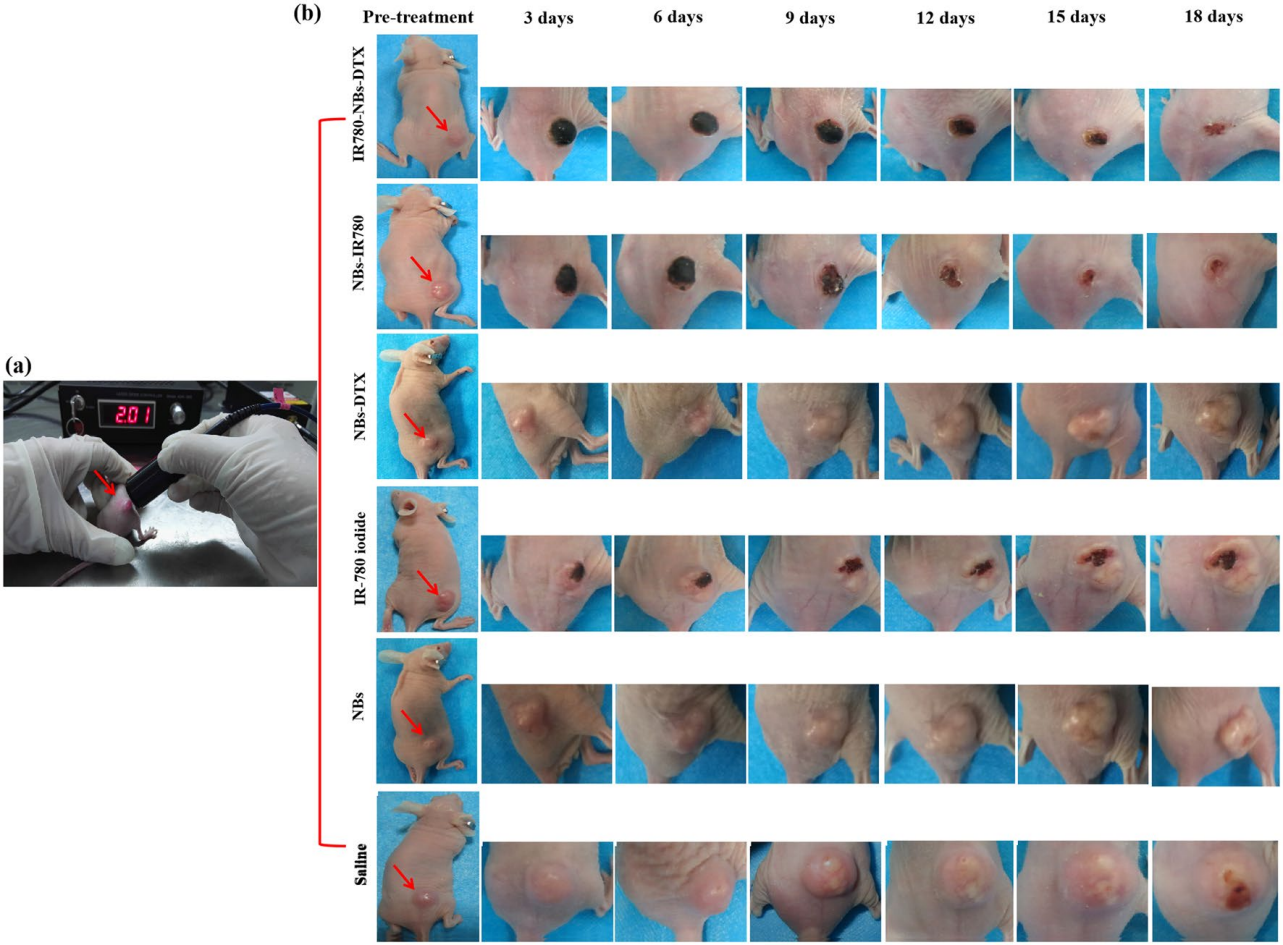
Photothermal ablation combined with chemotherapy mediated by IR780-DTX-NBs for pancreatic cancer xenografts. (**a**) Photothermal ablation of the tumor (indicated by red arrow) via a photothermal therapeutic instrument (1 w/cm^2^, 210 s). (**b**) Changes in tumor volume (indicated by the red arrow) after combined treatment with different contrast agents (IR780-NBs-DTX, NBs-IR780, NBs-DTX, IR-780 iodide, NBs, and saline).

**Figure 13:**
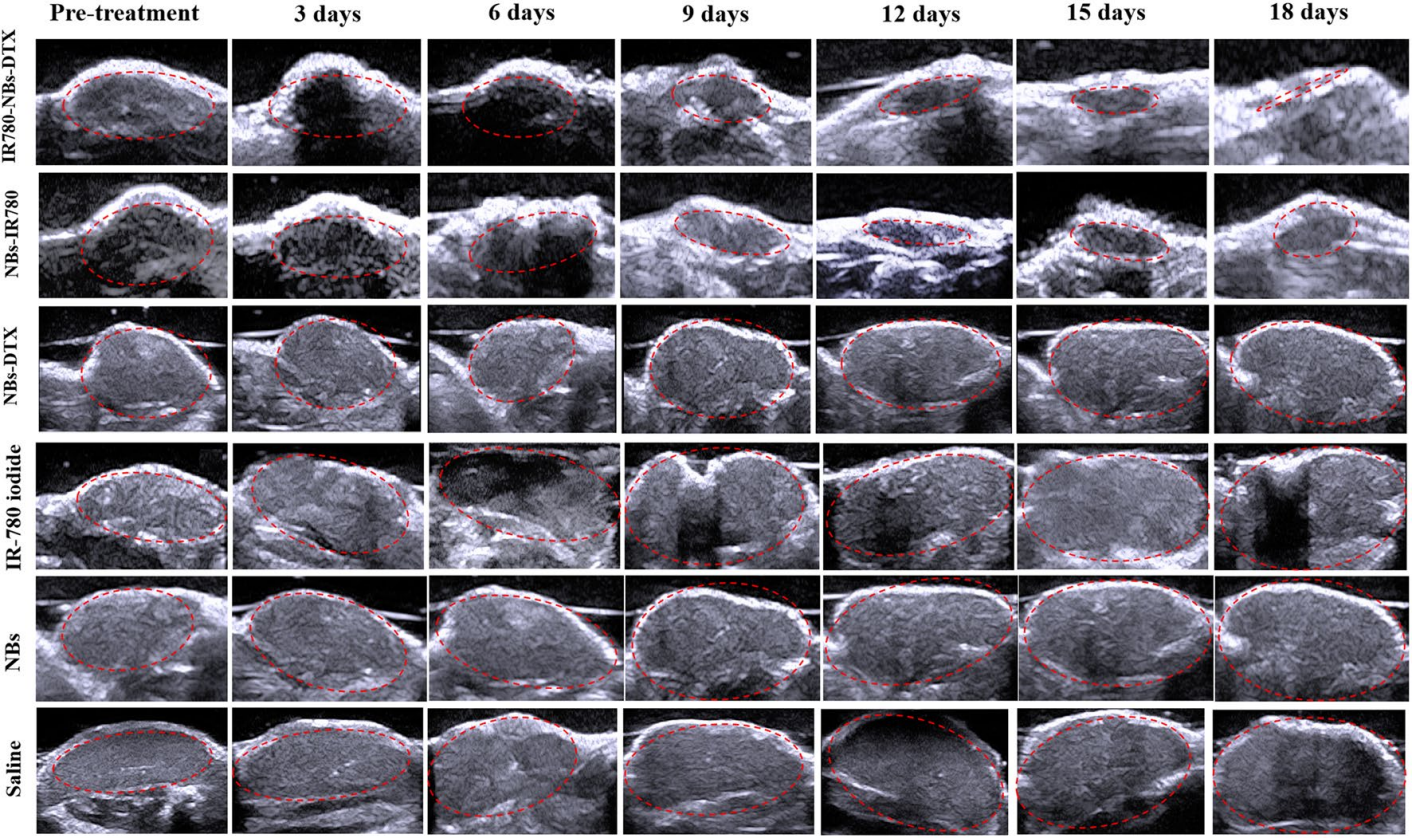
Changes in tumor volume were monitored by traditional ultrasound before and after combined treatment with different contrast agents (IR780-NBs-DTX, NBs-IR780, NBs-DTX, IR-780 iodide, NBs group, and saline). Red dotted lines represent tumors observed by ultrasound imaging.

**Figure 14:**
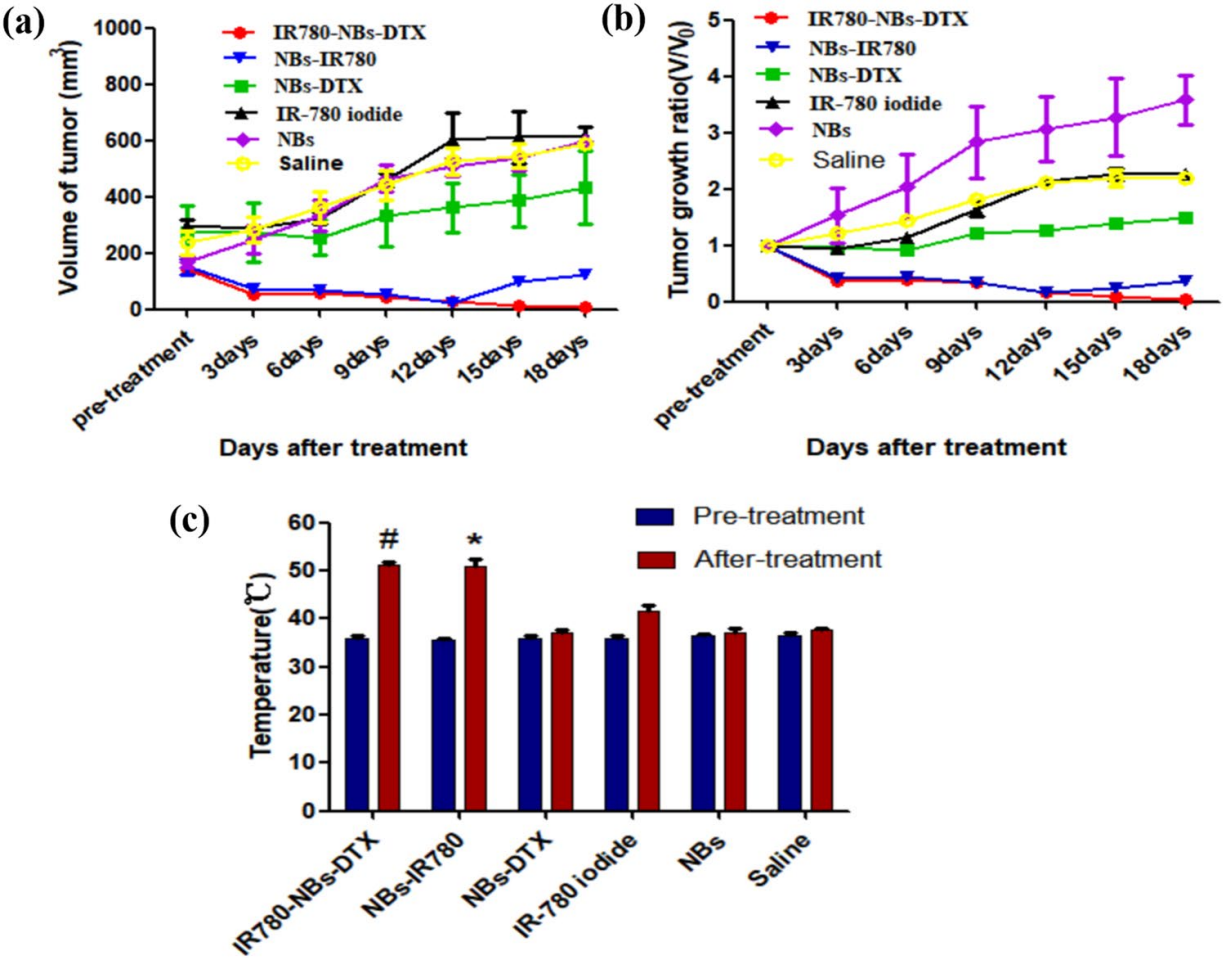
Statistical analysis of the changes in the volume and local temperature of pancreatic cancer xenografts in different groups before and after combined treatment. (**a**) The trend of tumor volume over time in different groups. (**b**) The trend of the tumor growth ratio (v/v0) over time in different groups. (**c**) The temperature change of pancreatic cancer in different groups before and after combined treatment. ^#^P < 0.05, significantly different from the temperature before combined treatment; *P < 0.05, significantly different from the temperature before combined treatment.

As shown in Figs. 12 and 13, in the IR780-NBs-DTX group, the tumor volume decreased from 3 to 15 days, and the tumors almost disappeared at 18 days. In the NBs-IR780 group, the tumor volume gradually decreased from 3 to 15 days, but the periphery of the tumors increased at 18 days. Then, in the NBs-DTX group, the tumor volume decreased at 3 days and 6 days; however, from 9 to 18 days, the volume gradually increased despite a slower rate than that of the IR-780 iodide group, NBs group and the saline group. Last, in the IR-780 iodide group, NBs group and saline group, from 3 to 18 days, the tumor volume gradually increased. The changes in tumor size and tumor growth ratio (v/v_0_, v: the tumor volume at different days after treatment; v_0_: the tumor volume before treatment) of every group were statistically analyzed in Fig. 14a,b.

In addition, to verify the photothermal effect mediated by IR780-NBs-DTX, the temperature of tumors before and after treatment in every group was examined. The results are shown in Fig. 14c. In the IR780-NBs-DTX group, the local temperature of the tumor increased from 36.7 ± 1.5 °C to 52.4 ± 1.2 °C before and after photothermal treatment, and the difference was statistically significant (P < 0.05). Meanwhile, in the NBs-IR780 group, the temperature changed from 36.2 ± 1.9 to 51.9 ± 1.8 °C, and the difference was statistically significant (P < 0.05). However, the local temperature of tumors increased slightly in the IR-780 iodide group, and almost no increase was observed in the NBs-DTX group, NBs group and saline group.

### Molecular level observation of the tumor therapeutic effect mediated by IR780-NBs-DTX ex vivo

**Figure 15:**
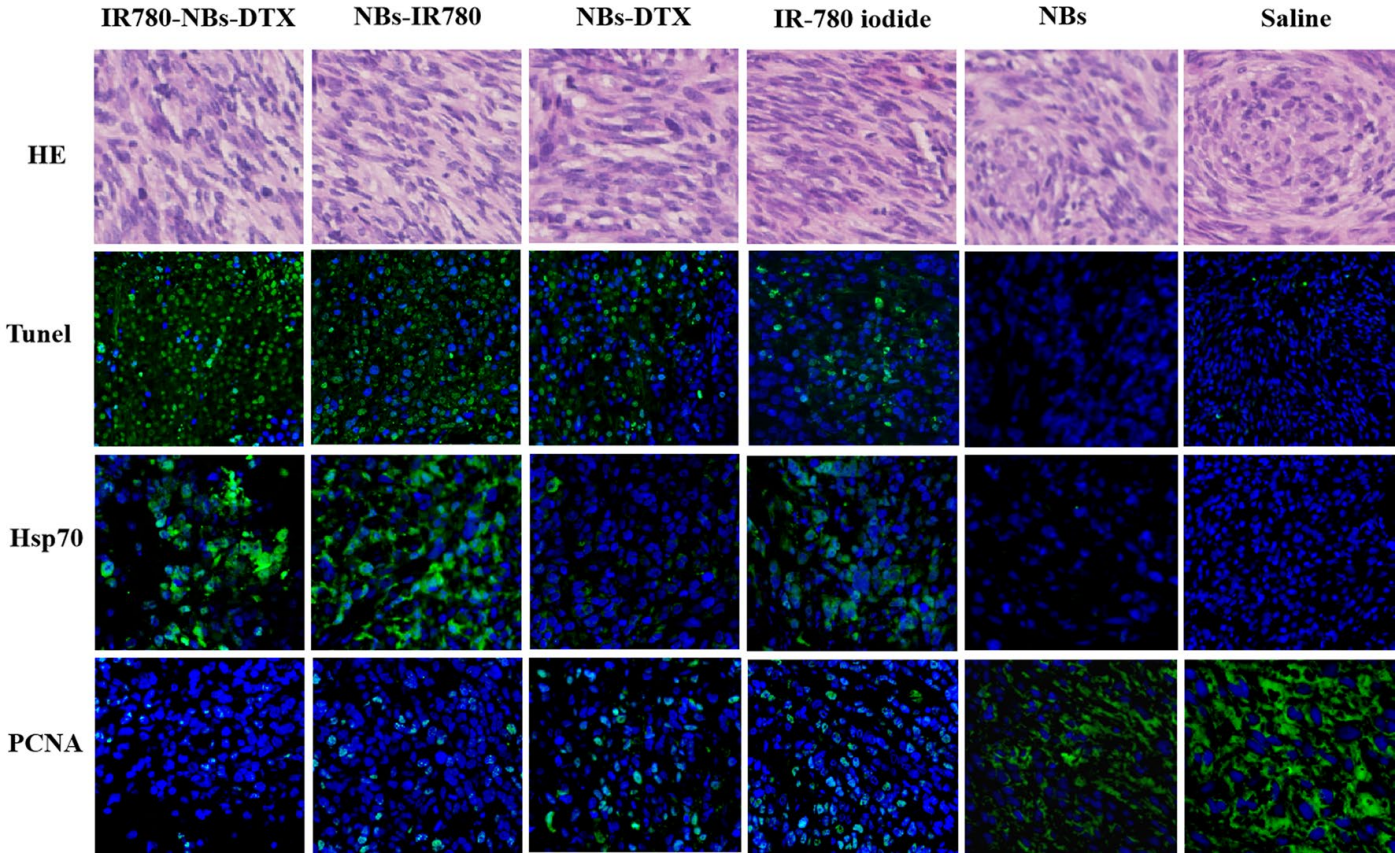
Molecular-level observation of the tumor therapeutic effect mediated by different contrast agents (IR780-NBs-DTX, NBs-IR780, NBs-DTX, IR-780 iodide, NBs, and saline) through immunohistochemistry and immunofluorescence ex vivo.

To further observe the therapeutic effect mediated by IR780-NBs-DTX for xenotransplanted pancreatic cancer, immunohistochemistry and immunofluorescence were performed. As shown in Fig. 15, all groups were verified by hematoxylin–eosin staining to have the same proliferation level of tumor cells. The apoptosis of tumor cells in the IR780-NBs-DTX group was the greatest compared with the other groups. Then, from the NBs-IR780 group to the saline group, the apoptosis of tumor cells gradually decreased. For Hsp70, there was high expression in the IR780-NBs-DTX group and NBs-IR780 group, whereas there was little expression in the IR-780 iodide group. There was almost no Hsp70 expression in the NBs-DTX, NBs and saline groups. Finally, PCNA expression in the IR780-NBs-DTX group was the lowest. Then, from the NBs-IR780 group to the IR-780 iodide group, PCNA expression gradually increased, and the highest expression was in the saline group.

## Discussion

Because of its late detection, poor suitability for surgery, frequent recurrence after surgery and resistance to radiotherapy and chemotherapy, pancreatic cancer has a poor prognosis^27^. Identification of a strategy that can efficiently detect the initial small lesions of pancreatic cancer and implement targeted therapy is urgently needed. In this work, we prepared multifunctional nanobubbles named IR780-NBs-DTX as UCAs; these NBs were used for pancreatic cancer diagnosis with dual-mode imaging and targeted treatment with photothermal therapy combined with chemotherapy.

First, the newly prepared IR780-NBs-DTX have good characteristics. Although different shell membranes of NBs were explored in previous studies^12^, lipids were selected in this research; as the shell membranes of NBs are more conducive to CEUI and IR-780 iodide and DTX have liposoluble properties and are more easily carried in lipid shell membranes^10^, the best formulations, with an initial dosage of 0.15 mg IR-780 iodide/1.0 mg DTX and an appropriate concentration (6 × 10^6^ bubbles/mL) of IR780-NBs-DTX for CEUI, were verified in this study. Importantly, the concentration (6 × 10^6^ bubbles/mL) is within the appropriate biosafety range according to Fig. 5c. In our previous study^10,18,24^, although different NBs were prepared for tumor prognosis, there were no chemotherapy drugs loaded in the NBs. In theory, loading more solid drugs may influence the size and elasticity of NBs, as well as the CEUI capability. As the thickness of lipid membranes may influence the particle size of NBs^10,21^, the results obtained in this study confirmed that the size of the lipid membranes was also related to the size distribution of IR780-NBs-DTX. When chemotherapy drugs are added to lipid membranes, it is difficult for the traditional membrane hydration method to reduce the particle size of MBs^24^, which was confirmed in our study. However, using high-speed shearing, the lipid membranes became very small, so the particle size of IR780-NBs-DTX was also controlled in a small and uniform range. In this study, IR780-NBs-DTX with a particle size of 300–400 nm were favored for reaching the tumor microenvironment^28^.

Although NBs can pass through tumor vessels via the EPR effect, the poor tumor selectivity of NBs in vivo was verified by some previous studies^13–15,29,30^. Park et al. suggested that for any nanodrug delivery system, tumor-selective delivery over nontargeted organs must first be demonstrated in vivo, rather than assuming that EPR effects are related to the size of the nanoparticles^31^. To achieve tumor selectivity, the traditional strategy is to bond antibodies to the carriers via a chemical linking method. In this study, a tedious chemical procedure for IR-780 iodide loading in the shell membranes of NBs was not required, and the newly prepared IR780-NBs-DTX were given a highly effective targeting function for tumors. The in vitro and in vivo experiments confirmed that IR780-NBs-DTX achieved the maximum targeting accumulation of tumor cells under the targeting guidance of IR-780 iodide (Figs. 6, 7, 10). In contrast, the NBs had little selectivity for tumor cells in vitro; in the in vivo experiments (Fig. 10), there were a few NBs accumulating at the tumor site, which must be attributed to the EPR effect^28^. It is worth mentioning that immune clearance by the RES may also play an important role in the process by which NBs target tumors^32^. Although IR-780 iodide has high tumor selectivity, less IR-780 iodide arrives at the tumor site, which may be attributed to its poor water solubility and rapid clearance in vivo^33,34^. The results indicated that IR-780 iodide should be loaded into the nanocarriers to play a role in this strategy.

Theoretically, the intensity of NIRF signals in each organ reflects the accumulation of IR780-NBs-DTX in the organs because of the relatively low or even lack of autofluorescence observed in ex vivo organs^10^. To determine the cutoff point of treatment time after injection of IR780-NBs-DTX, it is necessary to explore the distribution and metabolism of IR780-NBs-DTX in various organs of the body. The results of this research verified that most IR780-NBs-DTX targeted the tumor site at 1 h after injection via the caudal vein (Figs. 10c, 11). Therefore, laser irradiation of tumors in vivo was performed 1 h after injecting IR780-NBs-DTX. Photothermal ablation of tumors combined with chemotherapy mediated by IR780-DTX-NBs was verified in both in vitro and in vivo experiments (Figs. 8, 12, 13, 14).

Perhaps because of DTX, the number of apoptotic cells was slightly higher in the IR780-NBs-DTX group than in the IR780-NBs group. For DTX alone, there were a few apoptotic cells, which may be because DTX is limited by its poor water solubility and low selectivity for cancer^25^. Therefore, in the in vivo experiments, the DTX group was excluded because of its poor therapeutic effect within the safe dose range. In addition, because IR-780 iodide is also insoluble in water^35^, only a small amount of the IR-780 iodide in the cell culture medium reached the tumor cells for photothermal ablation.

In contrast to some other studies, in the in vivo experiments, the size change of the tumors in each group was carefully monitored by conventional ultrasound, and the results were more accurate than before. First, a cavitation effect of IR780-NBs-DTX after release of DTX and IR-780 iodide into the tumor interstitial space was observed. At the same time, the shock wave generated by the cavitation effect maybe also severely damages the tumor^36^. Compared with traditional drug administration, DTX showed significantly increased concentrations in the local tumor, and the side effects decreased significantly. Interestingly, during 15 days after treatment, there was almost the same treatment effect between IR780-NBs-DTX and NBs-IR780, and the tumor size gradually decreased. However, 18 days after treatment, the tumor volume suddenly tended to increase in the NBs-IR780 group. These results imply that DTX in IR780-NBs-DTX may participate in further chemotherapeutic clearance of residual cancer cells after photothermal ablation of tumors. Notably, in the NBs-DTX group, the tumor volume increased gradually after a transient decrease, but the tumor growth rate was significantly lower than that of the control group. The results indicated that loading DTX into the targeted NBs strongly increases the effective concentration of DTX at the tumor site; nevertheless, the combination with photothermal therapy will be more effective in killing tumors. De Melo-Diogo et al. proved that maintaining hyperthermia at 50 °C or above in tumors during photothermal therapy is optimal because such a temperature increase elicits irreversible damage to cells, resulting in necrosis^37^. In this research, after laser irradiation, the surface temperature of the IR780-NBs-DTX group and NBs-IR780 group was confirmed to be more than 50 °C. At the same time, in the experimental group containing IR-780, there were different degrees of black scabs on the tumor surface, but no black scabs were found in the control groups, which indicated that IR-780 played an essential role in mediating photothermal ablation.

In summary, the novel prepared IR780-NBs-DTX were demonstrated to be good multifunctional UCAs and to have potential advantages for dual-mode molecular targeting imaging and targeted photothermal ablation combined with chemotherapy for pancreatic cancer. Subsequent research should focus on reducing the clearance of NBs by the immune system in vivo and enhancing the killing effect on tumors.

## Materials and methods

### Materials

Powders of 1,2-distearoyl-sn-glycero-3-phosphoethanolamine-N-[(polyethylene glycol)-2000] (DSPE-PEG(2000)) and 1,2-dipalmitoyl-sn-glycero-3-phosphocholine (DPPC) with high purity were purchased from Avanti Polar Lipids Inc. (Alabaster, AL, USA). DTX and IR-780 iodide were purchased from Sigma-Aldrich (St. Louis, MO). Octafluoropropane (C_3_F_8_) was purchased from Kehao Biological Technology Co., Xi’an. Hoechst 33342 and propidium iodide (PI) were purchased from Beyotime Technology (Shanghai, China). Cell Counting Kit-8 (CCK-8) was obtained from Dojindo (Japan). All other chemical reagents were analytical reagents that did not require further purification.

### Preparation of IR780-NBs-DTX and exploration of the appropriate entrapment of IR-780 iodide and DTX

IR780-NBs-DTX were prepared based on the optimized thin-film hydration method, which was previously described by our group^10,26^. First, three samples of the same hybrid lipids (7 mg of DPPC and DSPE-PEG (2000)) with appropriate mass ratios were weighed and placed in 25-mL flasks. Next, 0.5 mg, 1.0 mg and 1.5 mg of DTX were added to the three flasks, and the mixtures were dissolved in 3 mL of chloroform. The flasks were gently shaken to thoroughly dissolve the mixtures and mix them evenly. Then, 25 μL, 50 μL and 75 μL of IR-780 iodide (2 mg/mL) were added to the above solution in turn. The mixtures were agitated lightly via a magnetic stirrer (5 min, 300 rpm), and then rotational evaporation was performed (55 °C, 135 rpm, 30 min) to obtain the dried thin film. Then, the film was hydrated with 3 mL of hydration liquid (glycerol: PBS = 1:9 (v/v)), and the mixture was treated with a high-speed dispersed homogenizer (10,000 rpm, 5 min) to prepare a uniform liposomal film suspension. Then, every suspension was divided into two vials sealed with a rubber cap, evacuated via a 50-mL syringe with a long and fine needle, and filled with C3F8 after vacuum pumping. Finally, all of the samples were oscillated for 90 s in a mechanical oscillator (Ag and Hg mixer, Xi'an, China) to form NBs and immediately placed on ice. The procedure was carried out in the absence of light.

The synthesis process of NBs (FITC) was the same as that described above. For the optical detection of NBs via confocal laser scanning microscopy (CLSM) and flow cytometry (FCM), FITC was also incorporated into the lipid shells of NBs.

To explore the appropriate entrapment of IR-780 iodide and DTX in the NBs, the three different IR780-NBs-DTX described above were collected through centrifugal technology at low temperature (2000 rpm, 5 min). Then, standard curves of IR-780 iodide and DTX were generated, and the drug contents in the suspensions were detected via high-performance liquid chromatography (HPLC). Finally, the entrapment efficiency (EE) and drug loading (DL) were calculated by Eqs. (1) and (2) as follows:

1 $${\text{EE}} = \left( {{\text{Mass of total IR-780/DTX}}{-}{\text{Mass of unentrapped IR-780/DTX}}} \right)/{\text{Mass of total IR-780/DTX}} \times 100\%$$

2 $${\text{DL}} = \left( {{\text{Mass of total IR-780/DTX}}{-}{\text{Mass of unentrapped IR-780/DTX}}} \right)/{\text{Mass of total liposomes}} \times 100\%$$

### CEUI of IR780-NBs-DTX in vitro

The CEUI capability of IR780-NBs-DTX in vitro was measured by an analytical setup (Fig. 2a). A latex glove fingertip containing 10 mL of degassed water was set in a degassed water bath with an ultrasound transducer on one side. Two hundred microliters of different concentrations of IR780-NBs-DTX suspensions (with an initial addition of 0.15 mg IR-780 iodide/1.0 mg DTX; the parent suspension was diluted 5 times: 1.2 × 10^7^ bubbles/mL, 10 times: 6 × 10^6^ bubbles/mL, 20 times: 3 × 10^6^ bubbles/mL) were injected into the latex glove fingertip in turn, and SonoVue and PBS were used as controls. The CEUI was recorded by a Mylab Twice Ultrasound System (Esaote, Italy) with a 7.5 MHz transducer.The concentration of the bubbles was controlled via a hemocytometer, and the number of bubbles counted was the same as the number of cells, and the concentration was calculated using the same cell-counting method.

### The characteristics of IR780-NBs-DTX

The experimental procedures were performed according to our previous experiments^24,38^. First, 2 μL of Dio (1.2 mg/mL) was added to 1 mL of SonoVue suspension to stain the microbubbles for 5 min. Then, the IR780-NBs-DTX suspension (with an initial addition of 0.15 mg IR-780 iodide and 1.0 mg DTX, diluted to 6 × 10^6^ bubbles/mL) and SonoVue were dropped on slide glasses and detected using CLSM (Olympus Fv1000, Japan) with a 100 × oil-immersion objective lens. At the same time, another two drops of the suspensions were examined by transmission electron microscopy (TEM, FEIT12, America). Then, 2 mL of the suspensions were examined with a particle size analyzer (DelsaNano, Beckman Coulter, USA) at 25 °C to analyze the distribution of size and zeta potential. The above experiments were repeated at least in triplicate.

### The stability and photothermal effect of IR780-NBs-DTX

One milliliter of IR780-NB-DTX suspension (6 × 10^6^ bubbles/mL) was stored at 4 °C, and the shift in size distribution was observed at 5, 10, 20, 40, 60 and 80 min using a particle size analyzer (DelsaNano, Beckman Coulter, USA) at 25 °C. At the same time, a drop of the sample was collected on a hemocytometer at 5, 10, 20, 40, 60 and 80 min, the number of IR780-NBs-DTX was counted using the WCIF ImageJ software program, and the concentration of IR780-NBs-DTX (bubbles/mL) was calculated using the same cell-counting method. Finally, 1 mL of IR780-NBs-DTX suspension with the same concentration was placed in five wells of the six-well plate, and another 1 mL of PBS was used in the control well. All suspensions were irradiated for 350 s at a distance of 1 cm from the liquid surface by an 808 nm laser with different irradiation intensities (0.2, 0.4, 0.6, 0.8 and 1 w/cm^2^). The temperatures of the suspensions were measured and recorded every 50 s. Finally, the temperature curve over time was generated.

The above experiments were repeated at least in triplicate.

### The biocompatibility of IR780-NBs-DTX

The experimental procedures were performed according to our previous experiments^24,38^^.^ The biosafety of IR780-NBs-DTX was examined via a Cell Counting Kit-8 (CCK-8) assay with pancreatic cancer Mia-Paca2 cells purchased from ATCC. Mia-Paca2 cells were incubated in 96-well plates at a density of 5000 cells per well and cultured with DMEM in a humidified atmosphere (5% CO_2_, 37 °C). After 24 h, the culture media were replaced with the same volume of fresh DMEM containing various diluted concentrations of IR780-NBs-DTX (an initial addition of 0.15 mg IR-780 iodide/1.0 mg DTX and dilutions of 6 × 10^5^ bubbles/mL, 6 × 10^6^ bubbles/mL, 6 × 10^7^ bubbles/mL, 6 × 10^8^ bubbles/mL, 6 × 10^9^ bubbles/mL and 6 × 10^10^ bubbles/mL) for 24 h. Each concentration was repeated in five wells. Then, 10 μL of the CCK-8 reagent was added to each well and incubated for 4 h. Finally, the Infinite F200 multimode plate reader (Tecan, Männedorf, Switzerland) was used to examine the absorbance of each well at 450 nm. The cell viability at various concentrations of IR780-NBs-DTX was calculated via the formula C = (A − A0)/(A1 − A0) × 100% (where C: the cell viability; A: the absorbance of experimental wells; A0: the absorbance of blank wells; and A1: the absorbance of control wells).

### Tumor-targeting capability of IR780-NBs-DTX on pancreatic cancer cells in vitro

The experimental procedures were performed according to our previous experiments^24,38^. Mia-Paca2 cells were cultured in confocal Petri dishes with DMEM containing 10% FBS and maintained in a humidified atmosphere of 5% CO_2_ at 37 °C. When the cells reached approximately 70–80% confluency on the bottom of the dishes, the DMEM was discarded, and the cells were washed with sterile 1 × PBS three times. One culture dish containing cells was filled with 100 μL (6 × 10^6^/mL) of sterile IR780-NBs-DTX, and another dish was filled with 100 μL of sterile IR-780 iodide solution (150 μg of IR-780 iodide added to 100 μL of PBS). The last two culture dishes containing cells contained 100 μL (6 × 10^6^/mL) of sterile NBs (FITC) and 100 μL of PBS as a control. All of the above dishes were incubated at room temperature for 30–40 min. Next, the dishes were washed gently with 1 × PBS three times. Then, 1 mL (1 mg/mL) of Hoechst 33,342 was added to all dishes and incubated for 15 min at 37 °C. After being gently washed with 1 × PBS three times, all of the above dishes were examined via CLSM. In addition, to further quantify the targeting of IR780-NBs-DTX to pancreatic cancer cells, FCM was performed. Mia-Paca2 cells were cultured in four 25 mL culture bottles as previously described. After reaching approximately 80% confluency, the cells were gently washed with 1 × PBS as previously described. Next, 500 μL of the IR780-NBs-DTX solution (6 × 10^6^/mL), IR-780 iodide solution (150 μg of IR-780 iodide added to 100 μL of PBS), NBs(FITC) solution (6 × 10^6^/mL) and PBS were added to the culture bottles. After incubation at room temperature for approximately 45 min and gentle washing with 1 × PBS three times, the cells were digested with trypsin and collected in sterile test tubes for FCM analysis. All of the abovementioned procedures were repeated three times and carried out in the absence of light using aluminum foil.

### Effect of photothermal ablation combined with chemotherapy with IR780-NBs-DTX on pancreatic cancer cells in vitro

Mia-Paca2 cells were cultured in confocal Petri dishes under the same conditions described above and divided into five groups. When the cells reached 80% confluency, the DMEM was discarded, and the cells were gently washed with sterile 1 × PBS at least three times. Then, 100 μL of sterile IR780-NBs-DTX solution (6 × 10^6^/mL), NBs-IR780 solution (6 × 10^6^/mL), IR-780 iodide solution (150 μg of IR-780 iodide added to 100 μL of PBS), DTX solution (1.5 mg of DTX added to 100 μL of PBS) and PBS were added into the dishes in turn. An 808 nm laser (1.08 w/cm^2^, 90 s) was used to irradiate all of the dishes. Then, 1 mL of DMEM was added to each dish, and the cells were cultured in a humidified atmosphere of 5% CO_2_ at 37 °C for 1.5 h. Then, the DMEM was discarded, and the cells were washed gently with sterile 1 × PBS three times. Next, 100 μL of propidium iodide (PI) (5 μg/mL) was added to every dish, and the cells were incubated in the dark for 15 min. Finally, the PI in every dish was discarded, and the dishes were washed three times with 1 × PBS. CLSM was used to examine all dishes to detect cell apoptosis. All of the procedures were repeated at least three times.

### Animal models

All nude mice (BALB/c, approximately 18 g) were housed in accordance with the Guide for the Care and Use of Laboratory Animals adopted by the National Institutes of Health and carried out in compliance with the ARRIVE guidelines, and all procedures were approved by the Institutional Animal Care and Use Committee at the Fourth Military Medical University. Mia-Paca2 cells were suspended in 200 μL of 1 × PBS and injected subcutaneously into the flanks of nude mice (5 × 10^6^ cells per mouse). All in vivo experiments were performed when the tumor diameter reached 0.8–1.0 cm.

### CEUI of IR780-NBs-DTX in heterotopic subcutaneously transplanted pancreatic cancer in vivo

First, the tumor-carrying nude mice (n = 6) were anesthetized using 100 μL of 1% sodium pentobarbital via intraperitoneal injection and placed on a plate for subsequent experiments. The Esaote Mylab Twice ultrasound diagnostic apparatus was used to perform imaging of the tumors; the ultrasound transducer was gently placed on top of the tumors, and ultrasonic transmission gel was used to fill the space between them. Then, 200 μL of IR780-NBs-DTX solution (6 × 10^6^/mL) was injected intravenously into the mouse through the tail vein, and the imaging modality was switched to nonlinear harmonic imaging mode. After 30 min, 200 μL of SonoVue was injected in the same manner as IR780-NBs-DTX, and all of the parameters were held constant. All of the data and videos were stored and used for statistical analysis. The time-intensity curve for each sample was generated, and statistical analysis was performed using the GraphPad Prism 5 software program.

### Tumor-specific targeting and NIRF imaging capability of IR780-NBs-DTX in heterotopic subcutaneously transplanted pancreatic cancer in vivo

The nude mice bearing subcutaneous xenotransplanted pancreatic cancer were divided into four groups (n = 3/group) and injected intravenously with 200 μL of saline, NBs(FITC) solution (6 × 10^6^/mL), IR-780 iodide solution (3 μg of IR-780 iodide added to 200 μL of PBS) and IR780-NBs-DTX solution (6 × 10^6^/mL) via the caudal vein. After approximately 1 h, all of the nude mice were anesthetized with isoflurane and placed individually in the darkroom of the IVIS Lumina II imaging station (Caliper Life Sciences, Hopkinton, MA, USA) to detect FITC fluorescence (excitation filter: 465, emission filter: GFP) or NIRF (excitation filter: 745, emission filter: ICG) at the tumor site. Although different fluorescent dyes were detected in this experiment, the detection scales of each group were the same, and only the fluorescence intensity of each group was compared.

### Microscopic observation of IR780-NBs-DTX targeting and penetrating tumor vessels via immunofluorescence

Tumor-bearing nude mice were randomly divided into three groups (n = 3/group), and 200 μL of IR780-NBs-DTX solution (6 × 10^6^/mL) was injected into each mouse via the tail vein. After 1 min, 5 min and 1 h, the nude mice in the three groups were sacrificed, and the tumors were extracted for frozen section examination. All of the frozen sections were incubated with Isolectin-b4 (1:800, Beyotime, Haimen, China) at 25 °C for 8 h. Next, the sections were washed three times using 1 × PBS for approximately 10 min. Then, the frozen sections were incubated with an anti-biotin secondary antibody (1:1000, Beyotime, Haimen, China) for approximately 2 h, and the sections were again washed three times with 1 × PBS for approximately 10 min. Last, the nuclei of tumor cells were labeled by fluorescence staining with DAPI.

### The biodistribution of IR780-NBs-DTX ex vivo

The experimental procedures were performed according to our previous experiments^24^. The tumor-carrying nude mice were randomly divided into four groups (n = 3/group), and all of the nude mice were injected with 200 μL of IR780-NBs-DTX solution (6 × 10^6^/mL) via the tail vein. After 5 min, 1 h, 12 h and 48 h, the tumor-bearing nude mice of groups 1, 2, 3 and 4 were sacrificed, and then, the tumor, heart, liver, spleen, lung, kidneys and muscle were collected for ex vivo NIRF imaging with an IVIS Lumina II imaging station (Caliper Life Sciences, Hopkinton, MA, USA). The excitation filter was 745, and the emission filter was ICG. All of the above data were stored, and statistical analysis was performed.

### Photothermal ablation combined with chemotherapy mediated by IR780-NBs-DTX for subcutaneous xenotransplanted pancreatic cancer in vivo

The tumor-bearing nude mice were randomly separated into five groups (n = 6/group). Then, the mice in groups 1, 2, 3, 4 and 5 were intravenously injected with IR780-NBs-DTX solution (200 μL/mouse, 6 × 10^6^/mL), NBs-IR780 solution (200 μL/mouse, 6 × 10^6^/mL), NBs-DTX solution (200 μL/mouse, 6 × 10^6^/mL), IR-780 iodide (200 μL/mouse, 3 μg of IR-780 iodide added to 200 μL of PBS), NBs (200 μL/mouse, 6 × 10^6^/mL) and saline (200 μL/mouse), respectively. After 1 h, all of the tumor sites of the nude mice were irradiated with low-intensity focused ultrasound (2.5 w/cm^2^, 10 s) and an 808 nm laser in turn (1 w/cm^2^, 210 s), and the distance between the tumor surface and transducer was approximately 1 cm. In addition, the temperature of the tumor site in every mouse was detected and recorded before and after photothermal therapy. Then, the tumor size before treatment and after treatment was examined by ultrasound imaging every two days and recorded. The tumor volume was calculated by the formula “length × width × height × π/6”.

### Microscopic observation of the combined targeted therapeutic effect of IR780-NBs-DTX on subcutaneous xenotransplanted pancreatic cancer via immunohistochemistry (IHC) and immunofluorescence (IF) ex vivo

First, two nude mice from each group were randomly selected, and the tumors were harvested. After thorough washing with PBS, the tumors were fixed in 4% formaldehyde and embedded in paraffin. The paraffin sections were examined by IHC to confirm that the proliferation and differentiation of tumor cells in each group were basically the same. Then, 18 days after treatment, the tumors of the above treatment groups were harvested and embedded in paraffin again. Then, TUNEL staining was used to examine tumor cell apoptosis, whereas proliferating cell nuclear antigen (PCNA) immunolocalization was used to assess tumor cell proliferation. At the same time, the Hsp70 of every tumor was also evaluated through IF. All of the paraffin sections were observed via CLSM.

### Statistical methods

Statistical analyses were performed using independent-sample t-tests and one-way analysis of variance (ANOVA). A 95% confidence level was used to determine the significance of differences between groups, and P < 0.05 was designated significant. All data are reported as the mean ± standard deviation (S.D.).
